# The instructional modality used for contextual vertical integration of anatomy influences cognitive load and performance during an operative interpretation task in undergraduate gynaecology students: evidence from a multi-centre cluster-randomised controlled trial

**DOI:** 10.3389/fmed.2026.1827733

**Published:** 2026-05-21

**Authors:** Mohammed Ismail-Khan, Md. Siddique Ahmed Khan, Jabeen Aslam, Ratna Kumari, Arwa Areej, Ramana Devi, Tallapaneni Sreekanth, Fateen Fatima Mazher, Mohammed Musheer, Porfyrios Korompelis

**Affiliations:** 1 School of Medicine, University of Sunderland, Sunderland, United Kingdom; 2 Department of Gynaecological Oncology, Northern Gynaecological Oncology Centre, Queen Elizabeth Hospital, Gateshead, United Kingdom; 3 Department of Physiology and Medical Education, Shadan Institute of Medical Sciences, Hyderabad, India; 4 Department of Biochemistry and Medical Education, Shadan Institute of Medical Sciences, Hyderabad, India; 5 Department of ENT, Peterborough City Hospital, Peterborough, United Kingdom; 6 Department of Physiology, Rangaraya Medical College, Kakinada, India; 7 Department of Physiology, Shadan Institute of Medical Sciences, Hyderabad, India; 8 Department of Obstetrics & Gynaecology, Shadan Institute of Medical Sciences, Hyderabad, India; 9 Department of Biochemistry and Research & Development, Shadan Institute of Medical Sciences, Hyderabad, India; 10 Department of Anatomy, Shadan Institute of Medical Sciences, Hyderabad, India; 11 Department of Community Medicine and Medical Education, Dr. VRK Women’s Medical College, Aziznagar, India; 12 Department of Biochemistry and Medical Education, Ayaan Institute of Medical Sciences, Moinabad, India

**Keywords:** anatomy education, cognitive load, dimensionality, gynaecology, operative surgery procedures/methods, surgical anatomy, undergraduate medical education, vertical integration (VI)

## Abstract

**Introduction:**

Operative interpretation is central to the educational value of theatre placements, yet students often struggle to apply preclinical anatomy to the operative setting. Evidence increasingly supports teaching anatomy in a direct operative surgical context, rather than as isolated foundational knowledge. Such integration may better prepare learners for operative interpretation, in which anatomical knowledge must be applied to dynamic intraoperative views. This study examined whether the modality used for contextual vertical integration (CVI) of anatomy influences cognitive load and performance during an operative interpretation task.

**Methods:**

In a multi-centre, three-arm, cluster-randomised superiority trial across three Indian medical colleges, tutorial groups of MBBS students received a 60-min CVI session on pelvic anatomy for total laparoscopic hysterectomy with bilateral salpingo-oophorectomy via cadaveric prosection (CP), interactive three-dimensional digital anatomy (I3DD), or slide-based instruction (SB). One week later, students viewed an operative video, completed a 14-item surgical anatomy assessment, and rated intrinsic, extraneous, and germane cognitive load using an adapted Krieglstein questionnaire. Linear mixed models accounted for clustering at the tutorial-group level, with institution included as a fixed effect. Secondary outcomes included perceptions of cognitive load for the anatomy CVI session and a Cost-Consequence Analysis.

**Results:**

Of 400 students taught, 394 consented, and 383 completed the primary outcome assessment. Significant intervention effects were found for intrinsic, extraneous, and germane cognitive load during operative interpretation, and for surgical anatomy performance (all *p* < 0.001). Compared with CP and I3DD, SB was associated with higher intrinsic load, higher extraneous load, lower germane processing, and poorer surgical anatomy performance. Effect sizes for SB versus CP/I3DD were large for extraneous and germane load and moderate-to-large for surgical anatomy performance. No significant differences were detected between CP and I3DD for operative-interpretation cognitive load or performance. Per-student costs were ₹621 for CP, ₹361 for I3DD, and ₹143 for SB.

**Discussion:**

Three-dimensional, spatially congruent modalities appear to support transfer to operative interpretation more effectively than two-dimensional instruction, likely by reducing representational mismatch and avoidable processing demands. Cadaveric prosection and interactive 3D digital anatomy produced similarly favourable downstream cognitive load and performance outcomes, suggesting that the educational benefit may lie chiefly in preserving representational dimensionality during CVI.

## 1 Introduction

### 1.1 Operative surgery in undergraduate medical education

Operative surgery constitutes an essential element of undergraduate medical training, with theatre placements being an indispensable part of surgical placements (1). The UK National Undergraduate Curriculum in Surgery states that all medical students should receive adequate surgical exposure, delivered through surgical placements that include operating theatre sessions and supervised participation (2). In India, the undergraduate competency-based curriculum expects students to observe and assist in operative procedures (3). The educational rationale for this extends well beyond the acquisition of technical skill, as operating theatre placements provide the setting in which students can contextualise surgical disease, perioperative decision-making, patient safety processes, and team-based practice.

The operating theatre is, however, frequently experienced by students as a demanding learning environment, requiring them to navigate the workflow constraints of theatre, the emotional intensity of surgery, and the social dynamics of a hierarchical multidisciplinary team (4, 5) in addition to the demands of fulfilling their learning objectives. Undergraduate theatre learning is typically framed as situated, experiential learning in which students primarily observe (and occasionally participate in limited ways) in authentic workplace activity; its educational value lies in helping students connect core science to applied surgical practice, rather than in preparing them for independent operative performance (1, 6). There is, therefore, a strong argument that the broader learning opportunities the theatre affords depend on operative interpretation: the student’s ability to make sense of the operative steps, the anatomy being encountered, and the surgical rationale as the procedure unfolds. Without this, those learning opportunities may be rendered largely inaccessible.

### 1.2 Role of anatomy in operative interpretation & contextual vertical integration

Anatomical knowledge underpins the interpretation of operative surgery (7, 8); without it, the student cannot meaningfully distinguish tissue planes, identify structures at risk, or follow the logic of an operative procedure. However, anatomy is usually taught early in medical schools, and evidence suggests that retention declines substantially over time. Even within integrated curricula, knowledge may fall by around 15% in 18 months (9). In some context areas, decline over shorter intervals may be greater, with as much as half over 10 months (10). Consistent with this, students commencing surgical and obstetric–gynaecological rotations demonstrate clinically important gaps in surgical anatomy knowledge (11).

Critically, preclinical anatomy is commonly delivered as a regional/system-based body of knowledge, rather than framed around operative tasks and decision points (12); as a result, what students retain may be difficult to transfer to the dynamic interpretive demands of surgery without explicit contextual reinforcement and vertical integration (13–15). This disconnect has prompted sustained calls for the vertical integration of anatomy across its key clinical applications, so that anatomical knowledge is revisited and reinforced when it becomes clinically and procedurally relevant (13, 15).

Several interventions that contextually integrate anatomy with operative surgery have shown improvement in students’ anatomical understanding and confidence in the operating theatre (7, 8, 16), supporting them to make sense of what they observe in the theatre (15). However, undergraduate anatomy teaching in theatre is inherently opportunistic and frequently constrained by the primacy of patient safety, time pressure, and the surgeon’s concurrent responsibility to manage operative flow and supervise postgraduate trainees, limiting the feasibility of sustained, explicit anatomical demonstration during live cases (1, 6, 13).

Hence, revisiting relevant anatomy before theatre exposure in later years of medical school is a more effective strategy for equipping students to interpret what they observe in theatre (17). We refer to this process as contextual vertical integration (CVI): *contextual* because anatomy is revisited in direct relation to procedures and pathology that students encounter, and *vertical integration* because disciplinary learning is connected across stages of the curriculum (18, 19). CVI therefore aspires to transdisciplinary integration, the highest level of Harden’s integration ladder, where disciplines are taught together beyond traditional academic boundaries to foster deeper learning (18, 19). Understanding how different teaching modalities support CVI requires a theoretical framework that accounts for the cognitive demands of learning.

### 1.3 Cognitive Load Theory

The Cognitive Load Theory (CLT) provides a useful framework for examining how instructional design shapes the cognitive demands of learning and, thus, understanding why different anatomy teaching modalities may differ in their effectiveness in preparing learners for operative interpretation.

The theory, developed by Sweller (20), is grounded in the limited capacity of working memory (21) and distinguishes three types of cognitive load: intrinsic cognitive load (ICL), which arises from the inherent complexity of the learning task and the degree of element interactivity within it; extraneous cognitive load (ECL), which is imposed by how information is presented or by irrelevant demands in the learning environment; and germane cognitive load (GCL), which reflects the mental effort dedicated to constructing and automating cognitive schemas in long-term memory (22–24). Recent developments in CLT recognise GCL as the construct representing germane processing in working memory to enable schema acquisition, rather than a load per se (24). This study continues to refer to this construct as GCL, in line with conventional terminology and instrument semantics.

Learning is optimised when ICL is managed by matching the learning content to the learner’s level of expertise, ECL is minimised, and working memory resources are freed for germane processing (represented as GCL), leading to the effortful construction of schemas that organise knowledge into retrievable, transferable structures (23, 25). If the summative effect of ICL and ECL exceeds the working memory capacity, there are no working memory resources left to facilitate schema acquisition through germane processing (25–27). Hence, CLT offers a practical framework for evaluating instructional design to improve learning.

### 1.4 Theoretical synthesis: Cognitive Load Theory & operative interpretation

The operating theatre imposes additional extraneous demands on working memory, including the unfamiliar environment, team dynamics, and the emotional intensity of real-time surgery (1, 28). For many students, however, the more fundamental challenge is operative interpretation: making sense of the steps, anatomy, and underlying rationale of a procedure as it unfolds (6). In the present study, operative interpretation was operationalised using a standardised laparoscopic operative video to provide a consistent, camera-mediated view of procedural anatomy across learners.

Because operative interpretation is a key learning objective of theatre placements, the operative procedure itself provides the immediate material from which students are expected to learn in the operating room (6). Accordingly, operative interpretation was conceptualised here not as a detached *post hoc* test of knowledge, but as a standardised video-based interpretive task modelling a core educational activity of theatre placements: making sense of operative anatomy and procedural logic as a procedure unfolds. Although performance was measured during this task, the cognitive load construct of interest concerned the mental demands of operative interpretation in this structured learning task rather than cognitive load arising from a conventional summative examination format.

Operative interpretation imposes ICL because it requires simultaneous processing of multiple interacting elements, including identifying structures, understanding spatial relationships, and following the logic of sequential surgical steps (19, 27). Although undergraduate students in the clinical years will already have studied the relevant anatomy, these schemas may not be readily accessible at the point of operative interpretation because of attrition (9, 10), and may not be organised in a way that supports immediate operative application. Reactivation and contextual reorganisation are therefore likely to be necessary.

Operative interpretation can also impose ECL when the procedure is presented in a way that makes it difficult to gain an overview, identify key structures, and recognise links between sequential steps and relevant anatomy, thereby increasing avoidable processing demands that do not directly contribute to learning (20, 23). ECL may be further increased when learners must map prior anatomical knowledge onto a visually complex operative field and integrate prior and present information in real time (29).

When both ICL and ECL are high, working-memory resources may be consumed by basic comprehension and orientation, leaving insufficient capacity for germane processing (25, 26). This may impede deeper engagement with the construction of transferable operative knowledge. Contextually aligned pre-operative anatomy teaching may help address both ICL and ECL by reactivating anatomical schemas around a specific procedure, reducing perceived complexity during operative interpretation, and lessening avoidable mapping demands. It may also free cognitive resources for germane processing. However, the extent to which pre-operative anatomy teaching achieves these effects is likely to depend not only on its contextual alignment with the procedure, but also on the representational and interactive properties of the teaching modality used to deliver anatomy.

### 1.5 Anatomy teaching modalities for contextual vertical integration

If CVI supports operative interpretation by reactivating and organising anatomical schemas, a practical question follows: which teaching modality most effectively promotes transfer of anatomical knowledge to the operative context?

Several modalities are used in anatomy education, and they differ in how anatomical information is represented, manipulated, and mentally transformed by the learner (30, 31). These modality-specific affordances are likely to shape the cognitive demands students face when they later apply anatomical knowledge to interpret an operative procedure, operationalised in this study using a laparoscopic surgical video. Accordingly, this study compares three commonly used approaches to CVI, i.e., cadaveric prosection, interactive three-dimensional digital anatomy, and slide-based instruction, to examine how each prepares learners for operative interpretation.

Cadaveric prosection presents anatomy in its natural three-dimensional (3D) form, preserving spatial relationships, tissue planes, and structural layering (32). This is particularly relevant to recognising anatomy in procedural contexts. Prosections allow students to examine anatomy *in situ*, providing high representational fidelity (32, 33), and can be prepared in advance to support paced, segmented teaching in a deliberate sequence (32). These properties may help learners build anatomical schemas in a form that approximates later operative application and may therefore reduce ECL during operative interpretation.

Interactive 3D digital anatomy platforms also preserve 3D spatial relationships while allowing structures to be explored from multiple viewpoints, supporting understanding of depth, planes, and relative positioning in ways that align with procedural interpretation demands (30, 31, 34). Their capacity for rotation, zooming, and selective display of regions of interest also supports pacing, segmentation, and structured teaching (30). These features may similarly support schema construction in ways that reduce ECL during operative interpretation.

Slide-based instruction, typically delivered through annotated two-dimensional diagrams and textbook images, remains widely used because it is inexpensive and readily scalable (30, 31). It can also be carefully sequenced to support paced, stepwise teaching (35). However, slide-based representations offer limited depth and layering and therefore lower representational fidelity. Learners may consequently need to invest additional working-memory resources to translate two-dimensional (2D) views into 3D spatial relationships in procedural contexts, which could increase ECL during operative interpretation.

Although CVI of anatomy within surgical education has been associated with improvements in learner confidence, satisfaction, knowledge, and performance in operative settings (7, 8, 13–17), the cognitive mechanisms underlying these benefits remain under-specified. Moreover, although anatomy teaching modalities have been compared extensively in terms of anatomy learning outcomes (30–36), they have not been evaluated for their downstream effects on cognitive load during subsequent operative interpretation. The present study therefore examines whether anatomy teaching modality differentially shapes learners’ cognitive load when interpreting a standardised operative procedure.

For experimental purposes, standardising the operative case across learners means that any between-group differences in perceived ICL and ECL are likely to reflect differences in prior anatomical preparation and schema availability rather than differences in the procedure itself. Under these conditions, ICL principally reflects the element interactivity inherent in operative interpretation, whereas ECL reflects avoidable processing demands arising from the need to follow and integrate information with existing schemas during the task.

### 1.6 Objective

This study examined whether the modality used for contextual vertical integration of pelvic anatomy, specifically cadaveric prosection, interactive 3D digital anatomy, or slide-based instruction, influences intrinsic, extraneous, and germane cognitive load and surgical anatomy performance during interpretation of a standardised total laparoscopic hysterectomy with bilateral salpingo-oophorectomy (TLH + BSO) video.

Secondary objectives included modality-related differences in instructional-session (anatomy teaching) cognitive load and a cost–consequence comparison of the three modalities.

## 2 Methods

### 2.1 Study design

This study was a multi-centre, three-arm, cluster-randomised controlled superiority trial conducted across three undergraduate medical colleges in India. The trial compared three instructional modalities for delivering surgically relevant pelvic anatomy required for the interpretation of operative laparoscopic total hysterectomy and bilateral salpingo-oophorectomy (TLH + BSO). All interventions delivered identical anatomical content mapped to constructs required for operative interpretation.

The unit of randomisation was the pre-existing small-group teaching cohort (tutorial group) within each institution; the unit of analysis was the individual student, with statistical adjustment for clustering at the tutorial group level. Clusters were randomised in a 1:1:1 ratio to receive the anatomical content via one of three modalities: cadaveric prosection, interactive 3D digital anatomy instruction, or slide-based anatomy instruction.

Cluster randomisation was selected to preserve curricular integrity. The teaching intervention was embedded within scheduled curricular delivery as part of the formal implementation of a vertically integrated Spiral Curriculum in Reproductive Health.

The primary outcomes were cognitive load during operative interpretation and surgical anatomy performance, both assessed one week after the intervention in the same tutorial clusters. Secondary outcomes included instructional-session cognitive load and a cost–consequence comparison of instructional modalities. The study also reports on the psychometric evaluation of the adapted cognitive load instrument for operative interpretation.

Ethical approval was obtained from the Institutional Ethics Committee of Shadan Institute of Medical Sciences (19/07/SIMS/IEC/2025), which provided central ethical oversight for all participating colleges under a shared administrative governance structure. Written informed consent was obtained from all participants prior to randomisation. Non-participation had no academic consequences.

### 2.2 Participants

This trial was conducted between December 2025 and January 2026 across three undergraduate medical colleges in India offering the Bachelor of Medicine and Bachelor of Surgery (MBBS) programme.

Eligible participants were final-year MBBS students. All participants had previously completed foundational anatomy instruction in their first year of study, delivered through traditional region-based cadaveric dissection. Obstetrics and Gynaecology (O&G) is assessed in the final year, and students had completed structured O&G rotations between the second and fourth years, with structured placements designed to provide comparable operative exposure across cohorts, as documented in logbooks.

All final-year students scheduled to attend the designated O&G tutorial sessions during the study period were eligible. There were no additional exclusion criteria. Participation in the research component was voluntary; all students attended the scheduled teaching session as part of the curriculum, irrespective of research participation.

Students were informed about the study prior to cluster allocation. Written informed consent for research data collection was obtained at the individual level before randomisation. Of the 400 students who attended the scheduled teaching sessions (College A: 150; College B: 100; College C: 150), 394 provided written consent. Six students declined participation in the research data collection. The one-week outcome assessment was completed by 383 consenting participants; outcomes could not be measured for 11 students who did not attend the scheduled assessment session.

### 2.3 Cluster structure

Clusters were defined as pre-existing small-group O&G tutorial cohorts within each institution. These pre-existing tutorial groups were formed before study initiation, according to usual institutional practice, to include a spread of prior academic performance and were not reconfigured for research purposes.

A total of 33 tutorial groups were included (College A: 12; College B: 9; College C: 12). Cluster sizes ranged from 11 to 13 students (mean approximately 12). Eleven clusters were allocated to each intervention arm (Table 1).

**Table 1:** Cluster composition and participant flow by institution.

Institution	Total students taught	Students consented	Students followed up	Number of existing clusters	Cluster size range	Clusters per intervention arm
College A	150	147	144	12	12–13	4
College B	100	100	98	9	11–12	3
College C	150	147	141	12	12–13	4
Total	400	394	383	33	11–13	11

Cluster size reflects the number of students per pre-existing tutorial group. Clusters per intervention arm denote the number of tutorial groups allocated to each of the three instructional modalities (Cadaveric prosection, interactive 3D digital anatomy and slide-based) within each institution.

### 2.4 Randomisation and allocation concealment

Within each institution, tutorial groups were randomly allocated in a 1:1:1 ratio to one of three instructional modalities. Randomisation sequences were prepared in advance by an administrator not involved in teaching or outcome assessment, using sealed, opaque, sequentially numbered envelopes. Equal numbers of envelopes corresponded to each intervention arm within each institution (Colleges A and C: four per modality; College B: three per modality).

**Figure 1:**
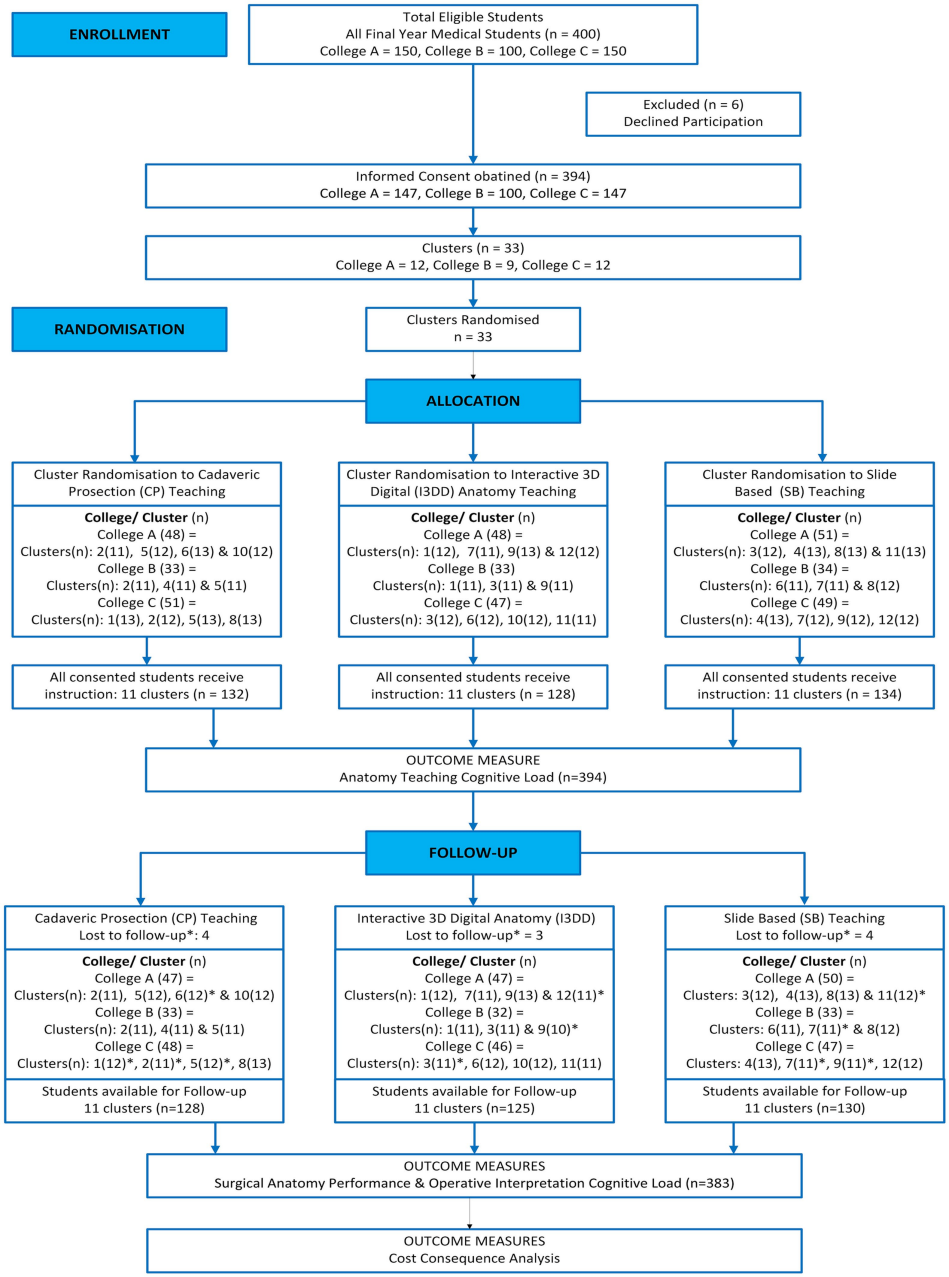
Across three undergraduate medical colleges, 400 final-year MBBS students were eligible and 394 provided written informed consent. 33 pre-existing O&G tutorial clusters were randomised in a 1:1:1 ratio to cadaveric prosection (CP), interactive three-dimensional digital anatomy (I3DD), or slide-based (SB) instruction. All randomised clusters received the allocated intervention, and all consenting students contributed to the immediate post-instruction cognitive load outcome (*n* = 394). At one-week follow-up, 383 consenting students completed the surgical anatomy assessment and operative-interpretation cognitive load measures. No clusters were lost to follow-up; losses occurred only at the individual student level. Asterisks indicate clusters in which at least one student was lost to follow-up. *n* denotes number of students.

Envelopes were opened one day prior to the scheduled teaching session to determine the assigned intervention for each tutorial group. Allocation was concealed from instructors until the envelope was opened. All randomised clusters received the allocated intervention. No clusters were lost to follow-up; individual-level loss to follow-up occurred for 11 consenting participants at the one-week outcome assessment. Participant flow through the trial is summarised in Figure 1.

### 2.5 Blinding and bias minimisation

Blinding instructors and students to the instructional modality was not feasible given the inherent nature of the educational interventions. However, several measures were implemented to minimise potential bias.

To reduce recruitment bias, informed consent was obtained at the individual level prior to cluster randomisation. Teaching was delivered as part of scheduled curricular activity, and all students attended their allocated session irrespective of research participation, thereby limiting self-selection effects.

To minimise performance bias, all teaching sessions across institutions and intervention arms were delivered by a single instructor using standardised lesson plans, instructional scripts, predefined learning objectives, and delivery checklists. This approach ensured consistency of content, sequencing, and emphasis across modalities.

Detection bias was addressed by anonymising surgical anatomy assessment scripts prior to marking. All scripts were scored by a single trained assessor who was blinded to intervention allocation and applied a predefined analytic scoring rubric. The use of a single blinded assessor was intended to maximise scoring consistency across institutions and intervention arms. Cognitive load instruments were self-administered under supervised conditions using standardised instructions. Analytical bias was minimised by conducting statistical analyses using coded group identifiers.

### 2.6 Sample size and statistical power

The number of clusters available for inclusion was fixed by the availability of intact tutorial groups scheduled within the participating institutions during the study period. The sample size was constrained by the number of intact tutorial groups available during the study period rather than by a conventional *a priori* calculation. However, before data collection, a design-stage detectable-effect analysis was undertaken to estimate the magnitude of between-group difference that the available cluster-randomised design would be able to detect. Sample size considerations accounted for clustering using the design effect:

Design Effect=1+(m−1)×ICC

where *m* represents the mean cluster size and ICC the intracluster correlation coefficient (ICC). Assuming a mean cluster size of 12 students and a conservative ICC of 0.05, consistent with estimates reported for educational interventions (37), the design effect was estimated at 1.55.

With 33 clusters and an anticipated total sample of approximately 396 students, the effective sample size after adjustment for clustering was estimated at approximately 255 participants. Under these assumptions, the available design provided 80% power at a two-sided *α* of 0.05 to detect a moderate standardised effect size (Cohen’s d ≈ 0.45) in planned pairwise comparisons of cognitive loads between instructional modalities.

The achieved primary outcome sample (*n* = 383) was close to the anticipated available sample and yielded an effective sample size of approximately 247 participants after adjustment for clustering. Thus, although the sample was constrained by the number of pre-existing tutorial groups and proceeded via omnibus intervention tests, the available design was sufficient to detect moderate between-group differences in the planned contrasts.

### 2.7 Development of a contextually vertically integrated surgical anatomy framework

Surgically relevant anatomical constructs required for accurate interpretation of TLH + BSO were identified through a structured two-round electronic Delphi consensus process. The expert panel comprised three gynaecology faculty members and three anatomy faculty members with expertise in pelvic anatomy teaching, ensuring balanced procedural and anatomical perspectives. Panellists independently generated anatomical constructs relevant to hysterectomy, which were collated and consolidated into a unified list. Constructs were rated using a 5-point Likert scale (1 = not important, 5 = essential) according to their importance for operative interpretation. *A priori* consensus criteria were defined as a median rating ≥ 4 with an interquartile range (IQR) ≤ 1. Following Round 1, anonymised, aggregated statistical feedback (median and IQR values) was circulated electronically, and constructs that did not meet consensus criteria were re-rated in Round 2 using the same thresholds. Consensus was achieved for all retained constructs after two rounds. The final construct framework was mapped directly to procedural stages of TLH + BSO and used to standardise instructional content and align assessment design across intervention modalities.

### 2.8 Interventions

All three instructional modalities delivered identical vertically integrated anatomical content derived from the Delphi-developed surgical anatomy framework. Teaching explicitly linked pelvic anatomical structures to the procedural stages of TLH + BSO, ensuring close alignment between anatomical instruction and operative interpretation. All sessions were delivered by a single instructor with experience in gynaecological laparoscopic surgery, which helped ensure that anatomical relationships were taught with explicit procedural relevance. The instructional objective across all arms was to enable students to identify, contextualise, and interpret surgically relevant anatomical landmarks within the context of hysterectomy.

Teaching was delivered in a small group format. Each session lasted 60 min and followed a standardised structure across modalities, including predefined learning objectives, a fixed instructional sequence mapped to procedural stages, and a structured discussion period for clarification.

In all intervention arms, teaching was instructor-led. During the structured discussion period, students were permitted to request clarification and direct attention to specific anatomical structures. Where applicable, students could indicate structures on the cadaveric specimen, guide manipulation of the digital model, or use a laser pointer to highlight structures on slide images. Total session duration and construct coverage remained identical across modalities.

#### 2.8.1 Cadaveric prosection arm

In the cadaveric arm, preserved abdominopelvic prosections were used to demonstrate surgically relevant anatomy in relation to procedural stages of laparoscopic hysterectomy. The specimen included pelvic organs and surrounding structures *in situ*, including bowel, kidneys, ureters, and bladder, permitting demonstration of spatial relationships and anatomical landmarks relevant to operative safety. A unilateral (left-sided) retroperitoneal dissection had been performed to expose the course of the ureter and its relationship to the uterine artery, while the contralateral broad ligament was preserved intact to demonstrate native anatomical planes. The same prosection model was prepared in advance and used consistently across all institutions to standardise anatomical exposure. The instructor identified key anatomical structures and explained their operative relevance in a didactic demonstration format.

#### 2.8.2 Interactive three-dimensional digital anatomy arm

In this arm, anatomical constructs were demonstrated using an interactive, screen-based 3D digital anatomical platform (3D Organon). The instructor used a tablet interface connected to a large display screen to dynamically manipulate anatomical structures, adjust viewing planes, and selectively add or remove surrounding systems (e.g., bowel, urinary tract, and vascular supply) to demonstrate relevant spatial relationships. Structures were visualised in relation to operative landmarks in accordance with the Delphi-derived construct framework. The instructional sequence mirrored that used in the cadaveric arm.

#### 2.8.3 Slide-based arm

In the slide-based arm, anatomical constructs were presented using static, high-resolution anatomical images and labelled diagrams delivered via structured slide-based instruction. Images were sequenced to demonstrate pelvic anatomy and spatial relationships, progressing in alignment with the procedural stages of laparoscopic hysterectomy. The instructor explicitly mapped each construct to its operative relevance through standardised narration. A digital marker function within the presentation software was used to highlight, trace, and emphasise key anatomical structures during explanation, allowing dynamic visual emphasis within the static 2D format. The instructional sequence and learning objectives were identical to those used in the other intervention arms.

#### 2.8.4 Ethical parity

To ensure ethical parity and avoid differential educational disadvantages, optional drop-in sessions that provided access to all three instructional modalities were offered to students after the completion of the study period. These sessions occurred after data collection and did not contribute to study outcomes.

### 2.9 Outcome measures

#### 2.9.1 Operative stimulus and operative-interpretation task context

At the one-week assessment session, participants viewed a standardised, de-identified TLH + BSO video from the institutional library under supervised conditions. The same edited 15-min video file was used across all institutions and included all procedural steps, with segments corresponding to Delphi-derived anatomical constructs, although these were not visually demarcated. The video was presented in 2D without audio using an HD projector under standardised viewing conditions across sites. Participants were seated to ensure an unobstructed view, playback was continuous without rewinding, and brief pauses were permitted only for completion of in-task responses. Room lighting was dimmed to optimise screen visibility. A 2D format was used because it reflects conventional viewing conditions for laparoscopic procedures in many undergraduate teaching settings, where 3D systems are not universally available.

TLH + BSO was selected as the operative stimulus because hysterectomy is both clinically common in the Indian context (38) and a core MBBS competency in the National Medical Commission curriculum under OG34.4 (Operative Gynaecology), for which videos are explicitly endorsed as an approved teaching–learning method (3).

The operative video depicted an extrafascial TLH + BSO for dysfunctional uterine bleeding in a patient with no previous abdominal surgery. This case was chosen to avoid pathological anatomical distortion, such as large fibroids, adhesions, malignancy-related infiltration, or prior surgical alteration, thereby allowing demonstration of standard pelvic anatomical relationships. The procedure followed the principles of conventional extrafascial dissection.

After completion of the video, surgical anatomy assessment, and cognitive load measures, all students participated in a structured debriefing session. Operative steps were revisited alongside an interactive 3D digital anatomical model (3D Organon) to reinforce anatomical relationships and consolidate the required NMC competency. This debrief took place only after outcome measurement and did not contribute to the study data.

#### 2.9.2 Primary outcome

The primary outcomes were cognitive load during operative interpretation (OI) of TLH + BSO (i.e., ICL_OI_, ECL_OI_, GCL_OI_) and surgical anatomy performance measured via a structured surgical anatomy assessment. All primary outcomes were assessed one week after the instructional intervention. Primary outcomes were evaluated in the subsequent tutorial session, within the same clusters as in the anatomy instruction.

Because all participants observed the same standardised operative case, the surgical steps and visual presentation were held constant across arms. Under this design, between-group differences in perceived ICL_OI_ were interpreted as reflecting differences in schema availability and the element interactivity of the task as experienced by learners (27), whereas ECL_OI_ reflected avoidable processing demands associated with following the operative presentation and integrating it with prior anatomical knowledge, including any split attention required to coordinate multiple sources of information during operative viewing (29).

During video viewing, participants completed a structured surgical anatomy assessment, with questions presented during relevant procedural stages to simulate real-time operative interpretation, consistent with theatre-based teaching practice. Sufficient time was provided during each procedural step to allow response completion. The assessment was structured according to the Delphi-derived construct framework. Questions were posed orally based on the surgical video, and students responded on paper scripts that also included lead-in questions. The assessment comprised 14 scored items with a maximum total score of 28. Each item was scored using a predefined analytic rubric, assigning 0 for incorrect or absent responses, 1 for partially correct responses, and 2 for fully correct responses. The full assessment is provided in Supplementary material 1. Total score was calculated as the sum of all item scores, with higher scores indicating greater anatomical understanding. All scripts were anonymised and scored by a single trained assessor who was blinded to intervention allocation.

Immediately following completion of the video and assessment task, participants completed the adapted 15-item Krieglstein Cognitive Load Questionnaire using a 9-point Likert response scale (39) (Supplementary material 2). The instrument comprises three theory-driven subscales representing ICL, ECL and GCL, each measured using five items. Subscale scores were calculated by summing the five constituent items, yielding a range of 5 to 45 per subscale. Higher ICL and ECL scores indicate greater perceived cognitive burden, whereas higher GCL reflects greater productive cognitive processing. Whilst acknowledging the recent changes in CLT, which do not view GCL as a “load” per se but rather as germane cognitive processing, the study retains the terminology of GCL, which aligns with the description of the psychological construct in the questionnaire used (24, 39). The primary analysis was based on participants who completed the one-week assessment (*n* = 383).

#### 2.9.3 Secondary outcomes

Cognitive loads during the anatomy teaching (AT) i.e., ICL_AT_, ECL_AT_ and GCL_AT_ were measured immediately following instruction using the original validated 15-item Krieglstein Cognitive Load Questionnaire (9-point Likert scale) (39). Subscale scoring was identical to that used for the adapted instrument, with possible scores ranging from 5 to 45 per subscale. This outcome was available for all consenting participants (*n* = 394).

#### 2.9.4 Sample validation of the cognitive load questionnaires

Krieglstein’s instrument was adapted to assess cognitive loads during operative interpretation. It preserved the original three-factor structure and response format, with item wording modified to explicitly reflect operative interpretation (39).

Internal consistency reliability was evaluated using Cronbach’s alpha for each cognitive load subscale (ICL_OI_, ECL_OI_, GCL_OI_), with coefficients ≥ 0.65 considered acceptable. Content validity was supported through the alignment of item wording for the operative interpretation task by faculty. Convergent validity was examined through correlations between the Paas 9-point Mental Effort Rating Scale (40, 41) and *a priori*-defined sums of the ICL_OI_ and ECL_OI_ subscales. This was in accordance with CLT where ICL and ECL conceptualise perceived cognitive difficulty, whereas GCL represents productive cognitive processing rather than a load on working memory (23, 24, 42). No exploratory factor analysis was conducted because the instrument’s structure was theory-driven and predefined.

Internal consistency for the Anatomy session for cognitive loads using original Krieglstein cognitive load subscales (ICL_AT_, ECL_AT_, GCL_AT_) was also examined in the present sample using Cronbach’s alpha, as a sample-specific reliability check. Convergent validity was similarly assessed by correlating the Paas Mental Effort Rating Scale (40, 41) with the combined ICL_AT_ and ECL_AT_ subscale scores.

#### 2.9.5 Administration and data handling

All instruments were administered in paper format under supervised conditions. Participants completed questionnaires independently using standardised written instructions, and discussion between participants was not permitted. Data were entered into Microsoft Excel prior to statistical analysis. The students assigned themselves an anonymisation code, which was consistently maintained on all questionnaires.

#### 2.9.6 Cost–consequence analysis

A cost–consequence analysis was conducted from the institutional perspective over the study delivery period (2025–26), with costs and outcomes presented in parallel rather than combined into a single summary ratio. Included costs were those relevant to modality choice: faculty and technician time, cadaver procurement and maintenance, personal protective equipment, and the 3D Organon software licence. Faculty personnel costs were included in full, as overall faculty costs varied across modalities because preparation requirements differed, although teaching delivery time itself was identical across arms. Common non-personnel costs such as teaching rooms and shared equipment were excluded. Cadaver procurement costs were apportioned at one-fifth, reflecting use of the abdominopelvic region of a single cadaver. Annual maintenance, repairs, and embalming costs for the 15-cadaver pool were first apportioned to one cadaver (1/15) and then to the abdominopelvic region of that cadaver (1/5). Per-student costs were presented alongside cognitive load and surgical anatomy performance outcomes. Full costing assumptions and calculations are provided in Supplementary material 3. No discounting was applied, given the time horizon of one year or less.

### 2.10 Statistical analysis

Analyses followed the principle of analysing participants according to their cluster’s allocated intervention (cluster-level allocation). Outcomes were analysed at the individual student level while accounting for cluster randomisation at the tutorial-group level. Primary analyses used complete cases at one week because missingness was low (<5%) and due to non-attendance. No equivalence or non-inferiority testing was planned; comparisons between arms were therefore interpreted descriptively using confidence intervals and standardised effect sizes rather than as evidence of equivalence.

#### 2.10.1 General modelling framework

All continuous outcomes were analysed using linear mixed-effects models (LMMs) in IBM SPSS Statistics (Version 31), estimated using restricted maximum likelihood (REML). Intervention arm and institution were specified as fixed effects. No intervention-by-institution interaction term was included, as the limited number of clusters per cell precluded stable estimation of such an effect. A random intercept for cluster group was included to account for cluster randomisation. The institution was modelled as a fixed effect given the small number of participating sites (*n* = 3). Degrees of freedom were estimated using the Satterthwaite method. If the random intercept variance approached zero (boundary estimate), fixed-effect estimates and associated inferences were still reported from the planned models, supported by the pre-specified cluster-aggregated sensitivity analysis.

ICCs were estimated from the variance components of the fitted LMMs using:

$$\mathrm{ICC}=\frac{\sigma_{\text{cluster}}^{2}}{\sigma_{\text{cluster}}^{2}+\sigma_{\text{residual}}^{2}}$$

Cognitive load subscales were derived from the sum of five 9-point Likert items (range 5–45) and were analysed as approximately continuous variables, consistent with established psychometric practice.

#### 2.10.2 Multiplicity control

Omnibus intervention effects were evaluated using linear mixed models. Where a significant omnibus effect was observed, pairwise comparisons between intervention arms were conducted using estimated marginal means with Bonferroni correction for the three pairwise contrasts.

#### 2.10.3 Effect size estimation

Standardised effect sizes were calculated in Python as Hedges’ g to provide small-sample bias correction. For each pairwise comparison, Cohen’s *d* was first computed by dividing the model-adjusted mean difference (*Δ*) by the pooled within-arm standard deviation. Hedges’ *g* was then obtained by multiplying *d* by the correction factor *J*:

$$SD_{\text{pooled}}=\sqrt{\frac{(n_{1}-1)SD_{1}^{2}+(n_{2}-1)SD_{2}^{2}}{n_{1}+n_{2}-2}}$$

$$g=\frac{\Delta }{SD_{\text{pooled}}}\times [1-\frac{3}{4(n_{1}+n_{2}-2)-1}]$$

where Δ is the model-adjusted mean difference between groups. The pooled standard deviation was calculated from observed (complete-case) within-arm standard deviations rather than model-derived estimates, so that effect sizes remain interpretable on the original measurement scale and are comparable with the wider cognitive load literature. Effect sizes were interpreted using conventional thresholds (small ≈ 0.2, moderate ≈ 0.5, large ≥ 0.8).

#### 2.10.4 Model assumptions and diagnostic strategy

Model assumptions were evaluated in SPSS (v.31) using residual diagnostics. Predicted values and residuals were saved from each LMM. Normality of residuals was assessed using Shapiro–Wilk tests, Q–Q plots, and histograms. Homoscedasticity was evaluated using residual-versus-fitted scatterplots and Levene’s tests (median-based) across intervention arms. Diagnostic results are reported alongside the findings in the Results.

#### 2.10.5 Sensitivity analysis

As a pre-planned sensitivity analysis, cluster-aggregated general linear models were conducted in SPSS (v.31) using tutorial-group means as the unit of analysis (*n* = 33 clusters), with intervention and institution as fixed factors and Bonferroni-corrected pairwise comparisons. This approach confirmed the robustness of findings to aggregation level, distributional assumptions, and the boundary variance estimates observed in the individual-level LMMs.

#### 2.10.6 Missing data

Missing data at the one-week assessment were due to non-attendance for outcome measures (*n* = 11; <5%). The distribution of missingness across intervention arms was examined using cross-tabulation. Given the low proportion of missing data and absence of meaningful imbalance across arms, complete-case analysis was undertaken for primary and secondary outcomes.

## 3 Results

### 3.1 Primary outcome: operative interpretation cognitive load (CL_OI_)

#### 3.1.1 Psychometric evaluation of the adapted operative interpretation cognitive load instrument

Internal consistency reliability was acceptable for all three adapted subscales (*n* = 383): ICL_OI_ (*α* = 0.75), ECL_OI_ (α = 0.786), and GCL_OI_ (α = 0.723). Convergent validity was supported by a strong positive association between Paas mental effort and the *a priori* combined ICL_OI_ and ECL_OI_ score (Pearson *r* = 0.73, *p* < 0.001; Spearman *ρ* = 0.74, *p* < 0.001).

#### 3.1.2 Operative interpretation cognitive load (CL_OI_)

A total of 383 students completed the one-week operative interpretation assessment and associated cognitive load measures. ICCs for ICL_OI_, ECL_OI_ and GCL_OI_ were negligible (≈ 0.00), indicating minimal between-cluster variability relative to within-cluster variability. For all three cognitive loads (CL_OI_), the estimated between-cluster variance was at the boundary of the parameter space (σ^2^_cluster ≈ 0); SPSS MIXED produced convergence warnings attributable to these boundary estimates, and the resulting models are therefore essentially similar to standard fixed-effects LMM. Results from the pre-specified LMMs are reported as planned. Across the three cognitive load outcomes, a statistically significant intervention effect was observed for ICL_OI_, ECL_OI_ and GCL_OI_ (all *p* < 0.001; Table 2).

**Table 2:** Operative interpretation cognitive load: estimated marginal means.

Cognitive load subtype	CP EMM (SE)	I3DD EMM (SE)	SB EMM (SE)	F (2, 378)	*p*
ICL_OI_	28.70 (0.38)	27.64 (0.39)	32.68 (0.38)	48.70	< 0.001
ECL_OI_	26.53 (0.42)	26.41 (0.43)	34.75 (0.42)	129.87	< 0.001
GCL_OI_	37.45 (0.47)	38.28 (0.47)	28.88 (0.46)	126.16	< 0.001

Estimated marginal means (EMM) and standard errors (SE) are derived from linear mixed models with cluster as a random effect. CP, cadaveric prosection; I3DD, interactive three-dimensional digital anatomy; SB, slide-based instruction; ICL, intrinsic cognitive load; ECL, extraneous cognitive load; GCL, germane cognitive load; OI, operative interpretation.

#### 3.1.3 Pairwise comparisons

Slide-based instruction was associated with significantly higher ICLOI compared with both cadaveric prosection and interactive 3D digital anatomy, with no statistically significant difference between the two.

ECL_OI_ was substantially higher in the slide-based group than in both cadaveric prosection and interactive 3D digital anatomy, which did not differ significantly from each other. Effect sizes were very large for slide-based comparisons (|*g*| ≈ 1.7; Table 3), indicating a substantial increase in non-productive cognitive burden under slide-based instruction as in Table 3.

**Table 3:** Operative interpretation cognitive load: Bonferroni-corrected pairwise comparisons.

Cognitive load subtype	Comparison	Mean difference (95% CI)	Bonferroni *p*	Hedges’ *g*
ICL_OI_	SB vs. CP	3.98 [2.69, 5.28]	< 0.001	0.89
	SB vs. I3DD	5.04 [3.74, 6.34]	< 0.001	1.12
	CP vs. I3DD	1.06 [−0.25, 2.36]	0.156	0.27
ECL_OI_	SB vs. CP	8.22 [6.80, 9.65]	< 0.001	1.68
	SB vs. I3DD	8.34 [6.91, 9.78]	< 0.001	1.76
	CP vs. I3DD	0.12 [−1.32, 1.56]	1.00	0.03
GCL_OI_	SB vs. CP	−8.57 [−10.14, −7.00]	< 0.001	−1.60
	SB vs. I3DD	−9.40 [−10.98, −7.82]	< 0.001	−1.80
	CP vs. I3DD	−0.83 [−2.42, 0.76]	0.629	−0.16

CP, cadaveric prosection; I3DD, interactive three-dimensional digital anatomy; SB, slide-based instruction; ICL, intrinsic cognitive load; ECL, extraneous cognitive load; GCL, germane cognitive load; OI, operative interpretation; CI, confidence interval.

Mean differences are presented as SB minus (CP or I3DD), or CP minus I3DD, as indicated. Bonferroni correction applied across pairwise comparisons within each cognitive load subtype. Hedges’ g interpreted as: small ≥ 0.20, moderate ≥ 0.50, large ≥ 0.80. A positive value indicates higher cognitive load in the first-named arm; a negative value indicates lower cognitive load in the first-named arm.

GCL_OI_, reflecting germane processing, was substantially lower in the slide-based group relative to both cadaveric prosection and interactive 3D digital anatomy, which again showed no significant difference between them (Table 3).

As seen in Table 3, effect sizes for slide-based comparisons were consistently large to very large (|*g*| = 0.89–1.80), whereas differences between the cadaveric prosection and interactive 3D digital anatomy were uniformly small (|*g*| ≤ 0.27).

#### 3.1.4 Model diagnostics and sensitivity analysis

Residual diagnostics for the operative interpretation LMMs indicated deviations from normality, as assessed by Shapiro–Wilk testing, for ICLOI, ECLOI, and GCL_OI_ (all *p* < 0.001). Levene’s test (median-based) suggested generally comparable residual variances across intervention arms for ECL_OI_ and GCL_OI_ (*p* = 0.314 and *p* = 0.233, respectively), with marginal evidence of heterogeneity for ICL_OI_ (*p* = 0.042). Given these distributional departures (and balanced group sizes; *n* ≈ 125–130 per arm), a cluster-aggregated general linear model (*n* = 33 clusters) was conducted as a sensitivity analysis to confirm robustness of inference. The intervention effect remained statistically significant for ICL_OI_, ECL_OI_ and GCL_OI_ (all *p* < 0.001), with pairwise conclusions largely consistent with those from individual-level LMMs. The sole exception was the ICL_OI_ comparison between cadaveric prosection and interactive 3D digital anatomy, which reached statistical significance in the cluster-level model (*p* = 0.002) but not in the individual-level LMM (*p* = 0.156), likely reflecting the aggregation of within-cluster variability. All other pairwise contrasts were unchanged in direction and statistical significance, confirming that results were stable to aggregation level and modelling approach.

### 3.2 Primary outcome: surgical anatomy performance

A total of 383 students completed the one-week surgical anatomy assessment. ICC was negligible (≈ 0.00), indicating minimal between-cluster variability relative to within-cluster variability. The estimated between-cluster variance was at the boundary of the parameter space (σ^2^_cluster ≈ 0), with convergence warnings as described above. A statistically significant intervention effect was observed: *F* (2, 378) = 25.14, *p* < 0.001 (Table 4).

**Table 4:** Surgical anatomy performance by instructional modality: estimated marginal means.

Surgical anatomy assessment	CP EMM (SE)	I3DD EMM (SE)	SB EMM (SE)	F (2, 378)	*p*
Surgical anatomy performance	21.96 (0.42)	22.08 (0.42)	18.44 (0.41)	25.14	< 0.001

Estimated marginal means (EMM) and standard errors (SE) are derived from linear mixed models with cluster as a random effect. CP, cadaveric prosection; I3DD, interactive three-dimensional digital anatomy; SB, slide-based instruction; surgical anatomy performance was measured on a standardised assessment.

#### 3.2.1 Pairwise comparisons

Slide-based instruction was associated with significantly lower surgical anatomy performance compared with both cadaveric prosection and interactive 3D digital anatomy, which did not differ significantly from each other. Effect sizes were moderate-to-large for both slide-based comparisons (|*g*| ≈ 0.72–0.74; Table 5), whereas the difference between cadaveric prosection and interactive 3D digital anatomy was trivial (|*g*| = 0.03; Table 5).

**Table 5:** Surgical anatomy performance: Bonferroni-corrected pairwise comparisons.

Surgical anatomy performance	Mean difference (95% CI)	Bonferroni *p*	Hedges’ *g*
SB vs. CP	−3.52 [−4.92, −2.12]	< 0.001	−0.72
SB vs. I3DD	−3.63 [−5.04, −2.22]	< 0.001	−0.74
CP vs. I3DD	−0.11 [−1.53, 1.30]	1.00	−0.03

CP, cadaveric prosection; I3DD, interactive three-dimensional digital anatomy; SB, slide-based instruction; surgical anatomy performance was measured on a standardised assessment; CI, confidence interval.

Mean differences are presented as SB minus (CP or I3DD), or CP minus I3DD, as indicated. Bonferroni correction applied across the three pairwise comparisons. Hedges’ g interpreted as: small ≥ 0.20, moderate ≥ 0.50, large ≥ 0.80. A negative value indicates lower performance in the first-named arm.

#### 3.2.2 Model diagnostics and sensitivity analysis

Residuals demonstrated approximate symmetry with deviation from normality on Shapiro–Wilk testing (*p* < 0.001). Levene’s test (median-based) indicated heterogeneity of variance across intervention arms (*F* (2, 380) = 7.59, *p* < 0.001), driven by greater dispersion in the slide-based group (SD = 5.49) relative to cadaveric prosection (SD = 4.13) and interactive 3D digital anatomy (SD = 4.21) arms. However, group sizes were balanced (*n* = 125–130 per arm), hence LMMs were considered robust to such variance inequality under balanced designs.

To confirm robustness, a cluster-level general linear model using aggregated cluster means (*n* = 33 clusters) was performed. The intervention effect remained statistically significant: *F* (2, 28) = 176.44, *p* < 0.001. Pairwise comparisons were identical in direction and statistical significance to the individual-level LMM, with both cadaveric prosection and interactive 3D digital anatomy outperforming slide-based instruction, with no significant difference observed between the two. The two, confirming that the surgical anatomy performance result is stable to aggregation level and variance assumptions.

### 3.3 Secondary outcome: anatomy teaching session cognitive load (CL_AT_)

#### 3.3.1 Psychometric evaluation of the anatomy teaching cognitive load instrument

Internal consistency of the instructional-session cognitive load subscales in the present sample was acceptable (*n* = 394): ICL_AT_ (*α* = 0.776), ECL_AT_ (α = 0.803), and GCL_AT_ (α = 0.78). Convergent validity was supported by a strong positive association between Paas mental effort and the combined ICL_AT_ and ECL_AT_ score (Pearson *r* = 0.73, *p* < 0.001; Spearman *ρ* = 0.76, *p* < 0.001).

#### 3.3.2 Anatomy teaching session cognitive load (CL_AT_)

A total of 394 students completed the anatomy teaching session and associated cognitive load measures. ICCs for ICL_AT_, ECL_AT and_ GCL_AT_ were negligible, indicating minimal between-cluster variability relative to within-cluster variability. A statistically significant intervention effect was observed for ICL_AT_, ECL_AT_ and GCL_AT_ (all *p* < 0.001; Table 6).

**Table 6:** Anatomy teaching session cognitive load by instructional modality: estimated marginal means.

Cognitive load subtype	CP EMM (SE)	I3DD EMM (SE)	SB EMM (SE)	*F* (2, 389)	*p*
ICL_AT_	32.37 (0.39)	29.59 (0.40)	34.42 (0.39)	38.20	< 0.001
ECL_AT_	33.23 (0.41)	31.01 (0.42)	35.56 (0.41)	30.81	< 0.001
GCL_AT_	38.33 (0.46)	37.97 (0.47)	31.87 (0.46)	63.34	< 0.001

Estimated marginal means (EMM) and standard errors (SE) are derived from linear mixed models with cluster as a random effect. CP, cadaveric prosection; I3DD, interactive three-dimensional digital anatomy; SB, slide-based instruction; ICL, intrinsic cognitive load; ECL, extraneous cognitive load; GCL, germane cognitive load; AT, Anatomy Teaching.

#### 3.3.3 Pairwise comparisons

ICL_AT_ demonstrated a graded pattern across modalities (Table 7), with interactive 3D digital anatomy lowest, cadaveric prosection intermediate, and slide-based instruction highest. All three pairwise comparisons were statistically significant, with effect sizes ranging from small-to-moderate (slide-based vs. cadaveric prosection, |*g*| = 0.44) to large (slide-based vs. interactive 3D digital anatomy, |*g*| = 1.05), and a moderate difference between cadaveric prosection and interactive 3D digital anatomy (|*g*| = 0.66).

**Table 7:** Anatomy teaching session cognitive load: Bonferroni-corrected pairwise comparisons.

Cognitive load subtype	Comparison	Mean difference (95% CI)	Bonferroni *p*	Hedges’ *g*
ICL_AT_	SB vs. CP	2.05 [0.73, 3.37]	< 0.001	0.44
	SB vs. I3DD	4.83 [3.50, 6.16]	< 0.001	1.05
	CP vs. I3DD	2.78 [1.44, 4.12]	< 0.001	0.66
ECL_AT_	SB vs. CP	2.33 [0.95, 3.71]	< 0.001	0.48
	SB vs. I3DD	4.54 [3.15, 5.94]	< 0.001	0.95
	CP vs. I3DD	2.22 [0.82, 3.61]	< 0.001	0.50
GCL_AT_	SB vs. CP	−6.45 [−8.00, −4.90]	< 0.001	−1.21
	SB vs. I3DD	−6.10 [−7.66, −4.54]	< 0.001	−1.14
	CP vs. I3DD	0.35 [−1.21, 1.92]	1.00	0.07

CP, cadaveric prosection; I3DD, interactive three-dimensional digital anatomy; SB, slide-based instruction; ICL, intrinsic cognitive load; ECL, extraneous cognitive load; GCL, germane cognitive load; AT, Anatomy Teaching; CI, confidence interval.

Mean differences are presented as SB minus (CP or I3DD), or CP minus I3DD, as indicated. Bonferroni correction applied across pairwise comparisons within each cognitive load subtype. Hedges’ g interpreted as: small ≥ 0.20, moderate ≥ 0.50, large ≥ 0.80. A positive value indicates higher cognitive load in the first-named arm; a negative value indicates lower cognitive load in the first-named arm.

ECL_AT_ demonstrated a similar graded pattern across modalities, with all three pairwise comparisons again statistically significant. Effect sizes were moderate for both slide-based versus cadaveric prosection (|*g*| = 0.48) and between cadaveric prosection and interactive 3D digital anatomy (|*g*| = 0.50), and large for slide-based versus interactive 3D digital anatomy (|*g*| = 0.95) as in Table 7.

GCL_AT_ was substantially lower in the slide-based group relative to both cadaveric prosection and interactive 3D digital anatomy, which did not differ significantly from each other (Table 7). Effect sizes for slide-based comparisons were large (|*g*| = 1.14–1.21), indicating markedly higher productive cognitive processing under both cadaveric prosection and interactive 3D digital anatomy compared with slide-based instruction.

#### 3.3.4 Model diagnostics and sensitivity analysis

Residual diagnostics for the instructional-session models indicated deviations from normality on the Shapiro–Wilk test across ICL_AT_, ECLA_T,_ and GCL_AT_ (all *p* < 0.001). Homogeneity of variance across intervention arms was supported by Levene’s tests (median-based) for all three cognitive loads (ICL_AT_ *p* = 0.876; ECL_AT_ *p* = 0.941; GCL_AT_ *p* = 0.392). Given the non-normal residual distributions (and balanced group sizes, n = 128–134 per arm), cluster-aggregated general linear models (*n* = 33) were performed as a sensitivity analysis to confirm the robustness of the findings. The intervention effect remained statistically significant for ICL_AT_ (*F* (2, 28) = 104.51, *p* < 0.001), ECL_AT_ (*F* (2, 28) = 83.21, *p* < 0.001), and GCL_AT_ (*F* (2, 28) = 196.19, *p* < 0.001), and pairwise contrasts were unchanged in direction and statistical significance. This confirms the robustness of findings to the modelling approach and aggregation level.

### 3.4 Secondary outcome: cost–consequence analysis

The itemised cost breakdown is presented in Table 8. Per-student costs were ₹621 for cadaveric prosection (*n* = 132), ₹361 for interactive 3D digital anatomy (*n* = 128), and ₹143 for slide-based instruction (*n* = 134). Cadaveric prosection was approximately four times more expensive per student than slide-based instruction, driven by cadaver procurement, maintenance, technician costs, and consumables. Interactive 3D digital anatomy was approximately twice the cost of slide-based instruction, with the differential attributable to the annual software licence (Table 9).

**Table 8:** Itemised cost breakdown by instructional modality.

Expense head	Cost component	CP	I3DD	SB	Basis
Faculty costs	Modality-specific preparation	₹9,600	₹3,600	₹3,600	₹1,200/h
	Delphi mapping and teaching design	₹2,400	₹2,400	₹2,400	₹1,200/h
	Teaching delivery (11 sessions)	₹13,200	₹13,200	₹13,200	₹1,200/h
Faculty subtotal		**₹25,200**	**₹19,200**	**₹19,200**	
Technician costs (CP only)	Prosection preparation (2 technicians × 6 h)	₹3,600	—	—	₹300/h
	Session facilitation (11 sessions × 1 h)	₹3,300	—	—	₹300/h
Technician subtotal		**₹6,900**	—	—	
Cadaveric material costs (CP only)	Cadaver procurement (1/5 apportioned)	₹26,000	—	—	1/5 of ₹1,30,000
	Annual maintenance and repairs (1/5 apportioned)	₹8,000	—	—	1/15 × 1/5 of ₹6,00,000
	Annual embalming chemicals (1/5 apportioned)	₹6,000	—	—	1/15 × 1/5 of ₹4,50,000
Cadaveric subtotal		**₹40,000**	—	—	
Consumables (CP only)	Personal protective equipment (11 sessions)	₹9,900	—	—	₹900/session
Technology costs (I3DD only)	3D Organon annual licence	—	₹26,955	—	$299.50 × ₹90
Total programme cost		**₹82,000**	**₹46,155**	**₹19,200**	
Cost per student		**₹621 (*n* = 132)**	**₹361 (*n* = 128)**	**₹143 (*n* = 134)**	

CP, cadaveric prosection; I3DD, interactive three-dimensional digital anatomy; SB, slide-based instruction. Cadaver costs apportioned at one-fifth of total procurement and maintenance expenses, reflecting the abdomen and pelvis as one of five standard dissection regions. Faculty costs calculated at ₹1,200 per hour; technician costs at ₹300 per hour. USD–INR conversion applied at ₹90 to the dollar (December 2025–January 2026). Costs common to all arms (teaching rooms, shared equipment) are excluded. Bold values indicate subtotal and summary cost rows, including subtotal costs, total programme cost, and cost per student.

**Table 9:** Cost–consequence summary: institutional costs and learning outcomes by instructional modality.

Outcome	CP	I3DD	SB
Costs (institutional perspective, 2025–26 INR)			
Total programme cost	₹82,000	₹46,155	₹19,200
Cost per consented studentᵃ	₹621	₹361	₹143
Primary outcomes: Operative interpretation cognitive load, EMM (SE)^b^			
ICL_OI_	28.70 (0.38)	27.64 (0.39)	32.68 (0.38)
ECL_OI_	26.53 (0.42)	26.41 (0.43)	34.75 (0.42)
GCL_OI_	37.45 (0.47)	38.28 (0.47)	28.88 (0.46)
Primary outcome: surgical anatomy performance, EMM (SE)^b^			
Surgical anatomy performance (/28)	21.96 (0.42)	22.08 (0.42)	18.44 (0.41)
Secondary outcomes: anatomy teaching cognitive load, EMM (SE)^c^			
ICL_AT_	32.37 (0.39)	29.59 (0.40)	34.42 (0.39)
ECL_AT_	33.23 (0.41)	31.01 (0.42)	35.56 (0.41)
GCL_AT_	38.33 (0.46)	37.97 (0.47)	31.87 (0.46)

Estimated marginal means (EMM) and standard errors (SE) are derived from linear mixed models with cluster as a random effect. CP, cadaveric prosection; I3DD, interactive three-dimensional digital anatomy; SB, slide-based instruction; ICL, intrinsic cognitive load; ECL, extraneous cognitive load; GCL, germane cognitive load; OI, operative interpretation; Surgical anatomy performance was measured on a standardised assessment with a score of 0–28; AT, Anatomy Teaching.

ᵃPer-student costs calculated using students who consented (CP = 132, I3DD = 128, SB = 134). ᵇPrimary outcomes assessed at one-week follow-up (*n* = 383: CP = 128, I3DD = 125, SB = 130). ᶜSecondary outcomes assessed immediately post-instruction (*n* = 394: CP = 132, I3DD = 128, SB = 134).

## 4 Discussion

To our knowledge, there is little experimental evidence on whether anatomy teaching modality influences cognitive load during subsequent operative interpretation, rather than anatomy learning outcomes alone. Both cadaveric prosection and interactive 3D digital anatomy led to significantly lower perceived ICL_OI_ and ECL_OI_, higher GCL_OI_, and better surgical anatomy performance during the interpretation of a standardised laparoscopic TLH + BSO compared with slide-based instruction. Effect sizes for these comparisons were consistently moderate to large (|*g*| range 0.72–1.80). Critically, cognitive loads experienced by students taught by cadaveric prosection and interactive 3D digital anatomy were not significantly different (|*g*| ≤ 0.27). These findings indicate that the representational properties of the anatomy teaching modality used for CVI have substantial downstream consequences for how learners process and interpret operative surgery, and that spatially rich, 3D modalities, whether physical or digital, confer a meaningful cognitive advantage over 2D slide-based instruction when the goal is to prepare students for operative interpretation.

### 4.1 Theoretical interpretation: why representational dimensionality matters

The significant differences in cognitive load between slide-based instruction and both cadaveric prosection and interactive 3D digital anatomy during operative interpretation are consistent with CLT. Interpreting an operative procedure requires learners to identify structures, appreciate spatial relationships, and follow sequential surgical steps, making it a task with high element interactivity (23, 27). The value of pre-session anatomy teaching therefore depends partly on how well the schemas formed during instruction match the representational demands of the operative context (22, 26).

Slide-based instruction encodes anatomy primarily in two dimensions. When learners then encounter a camera-mediated laparoscopic operative scene, they must reconstruct depth and layering and map these 2D representations onto a visually complex, dynamically unfolding procedure. This representational mismatch plausibly increases ECL by diverting working-memory resources towards spatial transformation and coordination rather than operative understanding. This interpretation is consistent with CLT accounts of increased workload when learners must integrate representationally disparate sources (23, 29), and with anatomy education research showing that 2D-to-3D translation and mental rotation can impose additional cognitive demands (30, 31, 43). The very large ECL_OI_ difference observed for slide-based instruction during operative interpretation (|g| ≈ 1.7) is consistent with this mechanism. At the same time, syntheses of 3D instructional approaches suggest that learning outcomes depend not only on dimensionality but also on the design quality and implementation of the resource itself (34, 44).

By contrast, cadaveric prosection and interactive 3D digital anatomy preserve three-dimensional spatial relationships during learning. Prosection does so through physical tissue *in situ*, whereas interactive 3D digital anatomy does so through rotatable models with selective layering. These modalities may therefore reduce representational mismatch at the point of operative interpretation by aligning how anatomy is learned with how it must later be applied. In CLT terms, this may reduce unnecessary mapping demands and other processing that does not directly support schema construction, thereby lowering ECL (22–24). This interpretation is consistent with work highlighting the value of prosection for spatial understanding in anatomy learning (32, 33) and with reviews suggesting that digital 3D anatomy can represent spatial structure more effectively than 2D resources (30, 31, 34), thereby supporting learning outcomes.

Although all groups interpreted the same standardised operative video, differences in ICL_OI_ were also observed. Within CLT, ICL reflects not only task element interactivity but also the learner’s available schemas (23, 25, 27). Learners taught with cadaveric prosection and interactive 3D digital anatomy may therefore have developed more coherent, contextually organised schemas, allowing the same operative segment to be processed as less complex through more efficient chunking of interacting elements (22, 25, 45). By contrast, less well-matched schemas after slide-based instruction may have increased perceived novelty, contributing to higher ICL_OI_ (|g| ≈ 0.89–1.12). This remains inferential, as schema structure was not measured directly, but it accords with the established relationship in CLT between schema availability and perceived ICL (23, 25).

The findings of GCL_OI_, representing the germane processing efforts of working memory (23, 24), provide complementary support. Cadaveric prosection and interactive 3D digital anatomy were associated with substantially higher GCL_OI_ than slide-based instruction (|*g*| ≈ 1.6–1.8). Within CLT, reducing extraneous demands can free working-memory capacity for productive processing: linking prior anatomical knowledge to the unfolding operative scene and forming or refining operative schemas (23–25). The concurrent improvement in surgical anatomy performance in the cadaveric prosection and interactive 3D digital anatomy groups is consistent with this account.

### 4.2 Similar downstream outcomes for cadaveric prosection and interactive 3D digital anatomy

Despite fundamental differences in representational medium, biological tissue with preserved spatial relationships and material properties (cadaveric prosection) versus a screen-based interactive 3D digital model produced closely similar downstream outcomes. During operative interpretation, the differences in ICL_OI_, ECL_OI_, and GCL_OI_ between these groups were small and non-significant (|*g*| ≤ 0.27), with confidence intervals spanning zero, and surgical anatomy performance was very similar (|g| = 0.03). Because this was a superiority trial with no pre-specified non-inferiority or equivalence margin, these results should be interpreted as an absence of detected differences rather than evidence of equivalence (46). Nevertheless, the pattern is consistent with evidence syntheses in anatomy education, indicating that digital 3D visualisation can achieve learning outcomes comparable to conventional approaches, including cadaver-based teaching, with effects varying by context and implementation, and potentially being more pronounced when spatial understanding is emphasised (31, 36).

A plausible explanation is that transfer to visually mediated operative interpretation depends less on biological realism per se and more on whether the instructional modality preserves 3D spatial relations and supports paced, structured exploration aligned to operative procedural stages. Preserving depth, layering, and relative positioning may reduce avoidable processing demands by limiting the need to mentally integrate disparate representations, thereby freeing working memory resources for schema construction and germane processing (24, 26). Both modalities in the present study were instructor-led and deliberately sequenced around procedural steps, and both present anatomically relevant information in a spatially integrated manner that should mitigate split-attention demands while benefiting from segmented, guided presentation of complex material (29, 47). Interactive 3D anatomy platforms (like 3D Organon) also support pan/zoom/rotation and layered structure control, enabling learners to interrogate spatial relations by selectively revealing and reorienting structures (31, 48).

This interpretation remains inferential because the study did not disentangle representational fidelity from instructional sequencing or interactivity, and alternative explanations cannot be excluded. Future work should compare CP and interactive 3D using a non-inferiority or equivalence design with an *a priori* meaningful margin in line with CONSORT guidance (46).

### 4.3 Graded pattern during anatomy teaching but convergence at operative interpretation

A key finding was that the cognitive-load profile observed during the anatomy teaching session did not fully reproduce the pattern seen one week later during operative interpretation. During anatomy teaching, the effects were graded: interactive 3D digital anatomy produced the lowest ICL_AT_ and ECL_AT_, cadaveric prosection was intermediate, and slide-based instruction was highest, with all pairwise differences statistically significant. GCL_AT_ during teaching was similar for cadaveric prosection and interactive 3D digital anatomy, and substantially lower after slide-based instruction. By the one-week operative-interpretation assessment, however, the differences between cadaveric prosection and interactive 3D digital anatomy had narrowed to small, non-significant values across cognitive load and surgical anatomy performance, whereas the slide-based group remained clearly inferior. Overall, this pattern suggests that cadaveric prosection and interactive 3D digital anatomy generated similarly usable schemas for the later operative task, despite differing during initial encoding.

Several non-mutually exclusive hypotheses may explain this graded-at-encoding but convergent-at-transfer pattern. First, an interface-supported segmentation and pacing hypothesis. Interactive 3D digital anatomy allows learners to control viewing angle, zoom, and structural layering, including selective addition or removal of structures (31, 48). These affordances may support learner-paced segmentation of complex visual information and reduce avoidable processing demands during initial encoding, consistent with CLT and multimedia principles of segmentation (23, 47). On this account, the teaching-phase advantage of interactive 3D reflects more efficient initial encoding, whereas cadaveric prosection may still support schema construction that is sufficient for later operative interpretation.

Second, a different encoding-routes hypothesis. Cadaveric prosection may impose higher immediate ICL_AT_ and ECL_AT_ than interactive 3D because learners must derive boundaries and 3D relationships from a visually complex biological specimen that includes partial occlusion and natural variability (49), rather than from a model that can present idealised views through selective display of layers and structures (48). Although prosections are often valued for their representational realism and their support of 3D understanding (33, 49, 50), experimental work on computer-generated anatomical visualisations suggests that greater realism can increase subjective cognitive load while still producing similar or better retention than more schematic renderings under some conditions (51, 52). From this perspective, the separation during anatomy teaching reflects higher representational processing demands during encoding, whereas the convergence at one week suggests that these different encoding routes may nonetheless yield comparably functional schemas for the downstream task.

Third, a threshold or task-sensitivity hypothesis. Both cadaveric prosection and interactive 3D digital anatomy may have enabled learners to reach a level of schema availability sufficient for interpreting the standardised operative video one week later, such that any remaining difference in schema richness was not detectable with this task or its measurement resolution. In CLT, once relevant schemas are available in long-term memory, working-memory demands during performance are reduced, and the incremental benefit of additional representational support may diminish (22, 23, 53). This interpretation should also be considered alongside possible measurement constraints, including ceiling effects or limited discrimination among higher-performing learners if the task is not sufficiently challenging or the scale has limited upper-end resolution (54). On this view, the one-week convergence indicates that both modalities produced schemas adequate for the level of transfer assessed, rather than that any earlier advantage had simply disappeared.

Fourth, contextual and affective influences may have shaped the reporting of cognitive load during cadaveric prosection. Exposure to the dissection hall, including the odour of formaldehyde, is commonly described as aversive and may be associated with sensory irritation or symptoms such as headache and nausea, with smell often identified as a trigger (55). Cadaver-based sessions can also provoke anxiety, particularly during early exposure (56). Because subjective cognitive load may be influenced by perceived stress and by the physical learning environment, such contextual discomfort could inflate reported load independently of instructional value (57). This would be consistent with cadaveric prosection showing higher reported ICL_AT_ and ECL_AT_ than the interactive 3D platform, while GCL_AT_ remained similar, suggesting that learners may have invested comparable germane effort but experienced greater situational and sensory demands during the session.

The present data cannot adjudicate among these explanations. Future work should test these possibilities more directly by manipulating specific instructional affordances, such as the degree of segmentation or selective structural revelation, to clarify the mechanisms underlying the observed teaching-phase differences and later convergence.

### 4.4 The 2D laparoscopic video as the operative-interpretation task context

Operative interpretation in this study was assessed using a standardised 2D, camera-mediated laparoscopic video rather than live observation in theatre. This was a deliberate methodological choice: use of a single video ensured that all participants across three institutions encountered the same operative case under identical visual conditions, thereby strengthening internal validity and allowing clearer attribution of between-group differences in the primary outcomes to prior anatomical preparation.

The 2D nature of the task warrants explicit consideration as both a limitation and an interpretive feature of the design. Evidence from laparoscopic simulation and training research suggests that 2D viewing is associated with higher perceived workload and poorer technical performance than 3D viewing (58, 59). Systematic review and meta-analytic evidence is broadly consistent with this, with 3D systems often linked to reduced error rates, shorter task or operative times, and more favourable subjective ratings (60, 61). Interpreting laparoscopic anatomy on a flat screen is therefore a demanding visuospatial task, requiring learners to infer depth, spatial relationships, and tissue planes without stereoscopic cues (58, 62). This likely increases reliance on indirect depth information and may impose additional extraneous demands on working memory.

At the same time, this task context helps interpret the findings. A flat, camera-mediated operative view likely increases dependence on the accessibility and spatial coherence of prior anatomical schemas: learners with stronger spatial schemas may compensate more effectively for absent depth cues, whereas those trained primarily with static 2D materials may face additional demands when reconstructing relations that were not fully specified during learning. This may help explain the higher ECL_OI_ observed at one week after slide-based instruction. More broadly, the surgical field of view in laparoscopy differs substantially from the views typically used to optimise anatomical understanding in any teaching modality, introducing an additional source of representational mismatch that may contribute to ECL during operative interpretation across all groups.

The screen-based format also offers a partial check against a simple format-congruence explanation. If a video-based task inherently favoured screen-delivered interactive 3D digital anatomy, clearer downstream differences from cadaveric prosection might have been expected. Instead, these two groups showed similar cognitive-load profiles and surgical anatomy performance during operative interpretation. Nevertheless, as a camera-mediated task, the video does not reproduce the full complexity of theatre-based learning (1, 6), so generalisation beyond video-based operative interpretation remains to be established.

### 4.5 Contextual vertical integration: educational, institutional, and equity implications

The present findings have implications beyond cognitive load and anatomy test performance. Our previous work showed that CVI can optimise cognitive load across undergraduate clinical O&G topics (63); the present study extends that work by showing that the modality used for procedure-aligned anatomy preparation may also shape cognitive load and performance during subsequent operative interpretation.

Undergraduate learning in the operating theatre is widely regarded as valuable but often unreliable. Learners may struggle with limited visibility, unclear learning goals, social hierarchy, theatre etiquette, and restricted opportunities for participation (1, 4–6). If students cannot follow the anatomical logic of the procedure, they are effectively excluded from much of the educational value of theatre placement. Prior work suggests that preparation and cognitive accessibility are key determinants of what students learn in theatre, including the ability to visualise the operation and understand its objectives (1, 6, 7). Specific findings in the theatre-learning literature illustrate this dependence on cognitive readiness: Fernando et al. reported that 30% of students felt unable to visualise the operation and considered this detrimental to learning; Lyon found that fewer than half of students felt the objectives for attending theatre were clear; and Zundel et al. identified advance knowledge of expected cases and learning goals as important supports for operating-room education (64–66). Taken together, this literature suggests that theatre learning depends heavily on learners entering with sufficiently developed schemas to interpret what they observe.

Against this background, the present results suggest that CVI modality meaningfully shapes the cognitive resources students bring to operative interpretation. Educationally, spatially richer anatomical preparation may do more than improve anatomy scores: it may reduce the cognitive burden of following the operation and thereby free working-memory resources (22–24), for broader learning in theatre, including perioperative decision-making, patient-safety considerations, team interactions, and professional role understanding (1, 6, 64).

At the teaching level, this also reinforces the importance of contextuality. Wider literature suggests that revisiting anatomy within an explicit clinical context improves students’ confidence and their ability to make sense of what they observe (7, 8, 15–17). More intelligible and inclusive theatre experience may also influence future interest in surgery, given that undergraduate surgical exposure can shape career intentions (67).

At the patient care level, the relevance of operative anatomical understanding extends beyond the theatre itself. Undergraduate exposure to surgery is important because surgical principles are encountered across medicine, including by doctors who do not ultimately enter surgical specialties (2). Moreover, operative interpretation extends beyond merely identifying structures. In procedures such as hysterectomy, understanding the ureter’s course relative to the uterine vessels and adjacent pelvic dissection planes helps learners appreciate why certain steps are carefully sequenced, where caution is required, and where procedural risk is concentrated. Although this study did not assess intraoperative decision-making directly, ureteric injury in gynaecological surgery illustrates the clinical importance of accurate operative-anatomical interpretation (68). Such understanding may also support perioperative care by prompting earlier consideration of ureteric injury in post-hysterectomy patients presenting with flank pain.

From an institutional perspective, where cadaveric facilities are available and sustainable, prosection remains a well-supported modality for preparing students to apply anatomy in clinical contexts (32, 33), including operative interpretation. Where cadaver access is structurally constrained, interactive 3D digital anatomy appears to offer a pragmatic alternative that produced similarly favourable downstream outcomes in this superiority trial at lower institutional cost. In our cost–consequence analysis, cadaveric prosection cost ₹621 per student, interactive 3D digital anatomy ₹361, and slide-based instruction ₹143; although slide-based teaching was the least costly, it was also associated with markedly poorer cognitive-load and performance measures for the outcomes assessed here.

At the level of global equity, the value of an effective non-cadaver modality lies not in diminishing cadaveric teaching, but in recognising that cadaver access is costly, regulated, and unevenly distributed, particularly in low- and middle-income settings settings (69). In the Indian context, barriers to body donation and cadaver availability remain substantial, including social, religious, and informational barriers (70). Scalable digital alternatives may therefore be especially relevant where cadaveric provision is limited or unreliable.

### 4.6 Methodological considerations, limitations, and future directions

This was a multi-centre cluster-randomised controlled trial across three institutions, with a large sample at the primary outcome (*n* = 383) and less than 5% loss to follow-up. Embedding the study within scheduled curricular delivery enhanced ecological validity. Its grounding in CLT offers mechanistic insight into why different instructional modalities may have produced different outcomes. All teaching was delivered by a single instructor using standardised Delphi-derived lesson plans, with explicit operative relevance supported by the instructor’s experience in gynaecological laparoscopy. Krieglstein’s cognitive load instruments (39) also underwent psychometric evaluation in the study sample, demonstrating acceptable internal consistency and strong convergent validity with Paas’ mental effort scale (40).

The number of clusters was constrained by the availability of intact tutorial groups embedded within scheduled curricular delivery rather than by a conventional *a priori* sample-size calculation. Nevertheless, a design-stage detectable-effect analysis indicated that the available sample was sufficient to detect moderate between-group differences. Future studies with more clusters per condition would be better powered to detect smaller effects and estimate cluster-level variance more precisely.

Operative interpretation was assessed using a standardised 2D laparoscopic video endorsed by the National Medical Commission for teaching operative gynaecological competencies (3). This ensured identical conditions across institutions, but it could not reproduce the full complexity of live theatre, where environmental, social, and real-time demands also shape learning (71). Evidence also suggests that 3D laparoscopic viewing may reduce mental workload relative to 2D viewing (58, 59), although comparison of video formats was beyond the scope of the present study.

During operative interpretation, participants also completed a structured surgical anatomy assessment at relevant procedural stages, introducing dual-task demands. Because this format was identical across study arms, it cannot account for the between-group differences in cognitive load. It was also designed to reflect theatre-based teaching practice, in which educators commonly question students during ongoing procedures to direct attention to key operative steps and relevant anatomy (72). The cognitive load scores should therefore be interpreted as reflecting a structured interpretive learning task rather than video observation alone. Their educational relevance in authentic theatre settings remains to be confirmed.

Cognitive load was measured by self-report, a widely used approach in cognitive load and multimedia learning research, with subjective instruments showing satisfactory reliability and validity (24, 73, 74). However, self-report captures perceived rather than objective load and may be influenced by prior experience or contextual factors (57). Future studies could complement subjective measures with physiological indices such as pupillometry or dual-task paradigms.

Use of a single instructor removed instructor variability as a confound but may limit generalisability. Multi-instructor studies, with instructor modelled as a random effect, would help determine whether the findings generalise across delivery contexts. The study also focused on a single procedure (TLH + BSO), selected for its clinical prevalence, alignment with National Medical Commission competencies (3), and suitability for demonstrating complex pelvic relationships. Generalisability to other procedures, anatomical regions, and surgical approaches remains to be established. Similarly, interactive 3D digital anatomy was delivered using a single platform (3D Organon), and outcomes may depend on design features, interface characteristics, and rendering quality (34, 44). Whether these findings extend to other interactive 3D anatomy platforms therefore requires further study.

Each modality also carries inherent representational limitations: cadaveric prosections require removal of overlying tissues, reducing fidelity to the layered surgical field; digital 3D models present idealised anatomy lacking biological variability and tissue texture; and static images cannot convey the anatomical variation encountered intraoperatively. These trade-offs were not isolated in the present design.

Residual diagnostics showed departures from normality across cognitive load outcomes, with marginal variance heterogeneity for ICL_OI_ and significant heterogeneity for surgical anatomy performance, driven by greater dispersion in the slide-based group. However, given the balanced group sizes, the LMMs were considered robust to these departures, and this was supported by cluster-aggregated sensitivity analyses, which reproduced the direction and significance of all primary contrasts.

Intracluster correlation coefficients were negligible across outcomes. This likely reflects the use of tutorial groups as the administrative unit of teaching delivery, which preserved curricular logistics while reducing within-session contamination by assigning all students in a session to the same modality and delivering sessions separately. It is also consistent with minimal between-cluster heterogeneity (37, 75), plausibly because tutorial groups contained students with varied prior academic performance. Although informal peer discussion between teaching and the one-week assessment cannot be excluded entirely, the cluster design minimised the main contamination pathway: within-session cross-arm exposure.

Finally, this was a superiority trial and was not powered to test formal equivalence between cadaveric prosection and interactive 3D digital anatomy. Although the consistent pattern of small, non-significant differences suggests similar efficacy, definitive conclusions would require a prospective equivalence or non-inferiority trial with a pre-specified educationally meaningful margin. This is an important next step in determining whether digital and cadaveric modalities are substitutable for achieving anatomical CVI in operative interpretation.

## 5 Conclusion

This study shows that the modality characteristics and dimensionality used for contextually vertically integrating anatomy have important consequences for subsequent operative interpretation. Although contextual alignment with the target procedure is essential, the effectiveness of this approach also appears to depend on how anatomical information is represented. Slide-based instruction was associated with higher extraneous cognitive load, lower germane processing, and poorer surgical anatomy performance during operative interpretation, whereas cadaveric prosection and interactive 3D digital anatomy were associated with more favourable cognitive load profiles and better performance. These findings suggest that preserving three-dimensional spatial relationships during contextual vertical integration better supports transfer to operative interpretation than two-dimensional instruction alone. In preparing students to make sense of operative surgery, modality is not merely a vehicle for content delivery; it forms part of the mechanism by which learning occurs.

## Data Availability

The raw data supporting the conclusions of this article will be made available by the authors, without undue reservation.

## Supplementary material

Supplementary material 1 Structured surgical anatomy assessment used during operative interpretation.

Supplementary material 2 Adapted operative-interpretation cognitive load questionnaire.

Supplementary material 3 Costing assumptions and calculations for the cost–consequence analysis.
