# Supplementary material for: The instructional modality used for contextual vertical integration of anatomy influences cognitive load and performance during an operative interpretation task in undergraduate gynaecology students: evidence from a multi-centre cluster-randomised controlled trial
Source: Front Med (Lausanne). 2026 May 21;13:1827733. doi: 10.3389/fmed.2026.1827733 (PMC13250826; doi:10.3389/fmed.2026.1827733)
Supplement: Supplementary material 1 — Structured surgical anatomy assessment used during operative interpretation. [file Supplementary_file_1.docx]

**Supplementary - 1**

**Surgical Anatomy Framework & Assessment Instrument**

*Total Laproscopic Hysterectomy + Bilateral Salpingo-Oophorectomy (TLH+BSO)*

**Document Sections**

- Part A — Comprehensive Anatomical Construct Framework for TLH+BSO
- Part B — Surgical Anatomy Assessment Questionnaire (14 items) Instructor Guide
- Part C - Surgical Anatomy Assessment Questionnaire (14 items) Student Version
- Part D — Scoring Rubric (Examiner Use Only)
- Part E — Construct-to-Question Mapping Matrix

| **PART A — Comprehensive Anatomical Construct Framework for TLH+BSO** |
| --- |

Constructs were identified via Delphi consensus and systematic procedural analysis. They are organised by anatomical constructs relevant within the operative steps if TLH+BSO

| **Anatomical Construct** | Detail / Operative Significance | Procedural Stage | **Domain** |  |
| --- | --- | --- | --- | --- |
| **Step 1: Initial Pelvic Anatomy** | | | |  |
| Uterus — macrostructure | Fundus, body, isthmus, cervix. The isthmus marks the level of uterine artery entry. The cervix, continuous with the vaginal vault, defines the plane of colpotomy. | All stages | Visceral |  |
| Uterine wall layers | Perimetrium (visceral peritoneum), myometrium, endometrium. Understanding these layers informs correct extrafascial dissection planes. | Anterior dissection; colpotomy | Visceral |  |
| Uterine cornu | Junction of uterine body with fallopian tube, round ligament (anteriorly), tube and utero-ovarian ligament. First operative landmark for ligament division. | Round ligament division | Visceral |  |
| **Step 2: BSO related Anatomy** | | | |  |
| Ovary | Intraperitoneal. Attached medially via utero-ovarian ligament and laterally via IP ligament. In mesovarium (posterior broad ligament leaf). First structure identified in the operative video. | Initial identification; BSO excision | Visceral |  |
| Fallopian tube | Interstitial → isthmic → ampullary → infundibular (fimbriae) segments. In mesosalpinx (upper border of broad ligament). Removed en bloc with ovary via IP ligament coagulation in BSO. | BSO component; IP ligation | Visceral |  |
| Infundibulopelvic (suspensory) ligament | | Peritoneal fold from ovary/tube to the lateral pelvic side wall. Contains the ovarian artery, ovarian vein, and lymphatics. Ligated for BSO. The ureter lies immediately medial and inferior - must be confirmed safe before energy activation. | BSO — IP ligation | Ligamentous / Support |
| Ovarian artery | | Direct branch of the abdominal aorta (below renal arteries). Descends retroperitoneally, crosses the ureter, and enters the pelvis in the IP ligament. Ligated during BSO. | IP ligament ligation (BSO) | Vascular |
| Ovarian vein | | Right drains into the IVC directly; left drains into the left renal vein. Both travel in the IP ligament and are ligated during BSO. | IP ligament ligation (BSO) | Vascular |
| Utero-ovarian (ovarian proper) ligament | | Cord-like structure connecting the medial pole of the ovary to the uterine cornu, immediately posterior and inferior to the fallopian tube. Distinct from and medial to the IP ligament. Divided during non-SO hysterectomies. | Non-SO hysterectomies | Ligamentous / Support |
| **Step 3: Parametrial Dissection related Anatomy (Part 1)** | | | |  |
| Broad ligament | | Anatomically a double-layered peritoneal fold (not a true ligament), extending laterally from the uterus to the pelvic side wall. Contains: fallopian tube (mesosalpinx), ovarian and uterine vessels, parametrial connective tissue, and nerves. | Broad ligament opening | Peritoneal |
| Round ligament | | Fibromuscular cord: uterine cornu → anterior leaf of broad ligament → deep inguinal ring → inguinal canal → labia majora. | First operative step | Ligamentous / Support |
| **Step 4: Anterior Dissection related Anatomy** | | | |  |
| Uterovesical peritoneal fold | | The anterior peritoneal reflection at the junction of the bladder and uterine body - a peritoneal structure, not a ligament. Incised to enter the avascular vesicocervical space, allowing safe inferior mobilisation of the bladder prior to colpotomy. | Anterior peritoneal entry | Peritoneal |
| Urinary bladder | | Lies anterior to the uterus and cervix; its base closely adheres to the lower uterine segment and supravaginal cervix. Mobilised inferiorly after incising the uterovesical fold. Trigone marks bilateral ureteric entry. Primary at-risk structure during anterior dissection. | Anterior dissection; bladder mobilisation | Visceral |
| **Step 5: Posterior Dissection related Anatomy** | | | | |
| Pouch of Douglas (rectouterine pouch) | | Deepest peritoneal recess in females. Bounded anteriorly by the posterior uterus/cervix, posteriorly by the rectum, and laterally by the uterosacral ligaments. Opened during posterior dissection for posterior parametrial division. Incorrect plane entry risks rectal injury. | Posterior dissection | Peritoneal |
| Rectum / rectosigmoid | Lies posterior to the uterus; forms the posterior boundary of the Pouch of Douglas. At risk during posterior peritoneal entry and uterosacral division if dissection strays outside the correct plane. | Posterior dissection; POD opening | Visceral |  |
| Rectovaginal space | | Avascular plane between the posterior vaginal wall and the anterior rectum. Entered during posterior dissection to allow rectal mobilisation away from the posterior vaginal wall prior to colpotomy. | Posterior dissection | Fascial Spaces |
| Uterosacral ligaments | | Paired condensations of endopelvic fascia running posterolaterally from the posterior cervix/upper vagina to the presacral fascia (S2–S4). Form the lateral boundaries of the Pouch of Douglas. Divided in posterior parametrial dissection. Classically incorporated in cuff closure for apical support. | Posterior dissection; cuff closure | Ligamentous / Support |
| **Step 6: Parametrial Dissection related Anatomy (Part 2)** | | | | |
| Ureter | | Retroperitoneal urinary tract structure (not a neural structure). Crosses the pelvic brim over the common iliac bifurcation → descends on the medial leaf of the broad ligament, lateral to the uterosacral ligament → passes immediately INFERIOR to the uterine artery ('water under the bridge') → traverses the parametrial tunnel of the cardinal ligament anteromedially → enters the bladder trigone. Four danger zones in TLH+BSO: (1) IP ligament ligation, (2) uterine artery ligation, (3) cardinal ligament division, (4) colpotomy. Must be confirmed safe before energy activation at each of these steps. | ALL parametrial steps — critical | Urinary Tract |
|  |  |  |  |  |
| Uterine artery | | Branch of the anterior division of the internal iliac artery. Runs medially in the cardinal ligament to the uterus at the isthmus, then ascends along the lateral uterine border, anastomosing with the ovarian artery at the cornu. Ligated at the isthmus. The ureter passes immediately inferior to it before entering the bladder. | Uterine artery ligation — key step | Vascular |
| Uterine vein(s) | | Accompany the uterine artery; drain into the internal iliac vein. Sealed simultaneously. Pericervical venous plexus within the cardinal ligament may bleed if the ligament is inadequately controlled. | Uterine artery ligation | Vascular |
| Cardinal (Mackenrodt's) ligament | | Transverse cervical ligament — the primary lateral support structure. Extends from the lateral cervix and upper vagina to the obturator fascia and pelvic side wall. Contains the uterine artery, uterine vein, and pericervical venous plexus. The ureter traverses its parametrial tunnel, passing immediately inferior to the uterine artery ('water under the bridge'). | Parametrial division; cuff support | Ligamentous / Support |
| **Step 7: Colpotomy and Vaginal Vault Closure Related Anatomy** | | | |  |
| Vagina / vaginal cuff | | Cervix projects into the vaginal lumen; circumferential colpotomy at the cervicovaginal junction detaches the specimen. The cuff is then closed; uterosacral and cardinal ligaments are classically incorporated in the apical suture bites to assist restoration of pelvic support. | Colpotomy; cuff closure | Visceral |
| Pericervical ring | | Fibrous ring at the cervicovaginal junction, receiving the cardinal, uterosacral, and pubocervical condensations. The colpotomy circumscribes this ring; cuff repair reconstructs support at this level. | Colpotomy; cuff closure | Pelvic Floor |
| Endopelvic fascia | | Connective tissue network condensing into named ligaments (cardinal, uterosacral) and fascia (pubocervical). Provides the structural framework for pelvic organ support. | Cuff closure | Pelvic Floor |

| **PART B — Surgical Anatomy Assessment Questionnaire** |
| --- |

| **Instructions to the Facilitator**  This document is the instructor and examiner version. It contains the full question text and the complete analytic scoring rubric for each item. It must not be shared with students prior to or during the assessment session.  **Before the session:**  **•** Ensure the standardised, de-identified TLH+BSO operative video file is loaded and tested on the projection system and the file marked SIMS 239_17/9 is used at all institutions for all sessions.  **•** Distribute Part C (Student Response Sheets) face-down before students are seated.  **•** Ensure students have pens. No additional reference materials are permitted.  **•** Dim room lighting to optimise screen visibility, consistent with standard laparoscopic viewing conditions.  **During the session:**  **•** Play the video continuously. Do not rewind.  **•** Pause the video at the segment timestamp shown for each question. Read the question aloud clearly, or instruct students to refer to their response sheet.  **•** Allow sufficient writing time at each pause before resuming playback. For two-part questions (Q9/Q10, Q2/Q3) ensure both questions are asked before resuming.  **•** While talking through the script, do not provide anatomical hints, corrections, or feedback during the session.  **After the session:**  **•** Collect all response sheets immediately and remind students to mention the anonymisation code on their scripts. |
| --- |

| **Segment 1** 00:31 │ *Video shows: Ovary* | |
| --- | --- |
| **Q1** | **Identify the structure demonstrated in this segment of the video.** |

| **Segment 2** 00:38 │ *Video shows: Infundibulopelvic ligament (adjacent to ovary)* | |
| --- | --- |
| **Q2** | **Identify the ligament demonstrated in this segment.** |

| **Segment 2** 00:38 │ *Video shows: Infundibulopelvic ligament (continued)* | |
| --- | --- |
| **Q3** | **Describe the vascular structures contained within this ligament and explain why precise identification and ligation of this structure is important during oophorectomy.** |

| **Segment 3** 01:26 │ *Video shows: Round ligament being divided* | |
| --- | --- |
| **Q4** | **Identify the structure being divided. Describe its anatomical course.** |

| **Segment 4** 02:06 │ *Video shows: Anterior leaf of broad ligament being opened* | |
| --- | --- |
| **Q5** | **Identify the peritoneal structure being opened. Describe its composition and list the structures it contains.** |

| **Segment 5** 03:00 │ *Video shows: Bladder in relation to lower uterine segment* | |
| --- | --- |
| **Q6** | **Identify the pelvic organ visible. Describe its anatomical relationship to the uterus at this stage of the procedure and explain why this relationship is surgically important.** |

| **Segment 6** 03:11 │ *Video shows: Uterovesical peritoneal fold being incised* | |
| --- | --- |
| **Q7** | **Identify the peritoneal fold being incised. Explain the purpose of this incision** |

| **Segment 7** 05:38 │ *Video shows: Anterior dissection plane during bladder mobilisation* | |
| --- | --- |
| **Q8** | **During this stage of anterior dissection, identify the structure requiring protection and explain the anatomical basis for its vulnerability in this plane.** |

| **Segment 8** 07:25 │ *Video shows: Pouch of Douglas (rectouterine pouch)* | |
| --- | --- |
| **Q9** | **Identify the anatomical space and describe its boundaries.** |

| **Segment 8** 07:25 │ *Video shows: Pouch of Douglas (continued)* | |
| --- | --- |
| **Q10** | **If posterior dissection proceeds outside the correct anatomical plane in this region, which adjacent structure is at greatest risk of injury? Explain why.** |

| **Segment 9** 08:10 │ *Video shows: Vessel being sealed at level of uterine isthmus* | |
| --- | --- |
| **Q11** | **Identify the vessel being sealed in this segment.** |

| **Segment 10** 08:56 │ *Video shows: Uterine artery in relation to cardinal and uterosacral ligaments* | |
| --- | --- |
| **Q12** | **Explain why medial positioning of the surgical instrument during sealing of this vessel is critical for operative safety based on its anatomical relationships.** |

| **Segment 11** 10:27 │ *Video shows: Cardinal (Mackenrodt's) ligament complex* | |
| --- | --- |
| **Q13** | **Identify this ligament complex. Describe its functional role in pelvic support.** |

| **Segment 12** 13:00 │ *Video shows: Vaginal vault transfixation following colpotomy* | |
| --- | --- |
| **Q14** | **During closure and transfixation of the vaginal vault, which named anatomical structures are being incorporated in the apical suture? Explain why their inclusion supports long-term pelvic organ support.** |

| **PART C — Surgical Anatomy Assessment Questionnaire** |
| --- |

| **Instructions to the Student**  You will watch a video of a Total Laparoscopic Hysterectomy + Bilateral Salpingo-Oophorectomy (TLH+BSO). The video will be paused at 12 points; at each pause, answer the question(s) on your response sheet.   - Write clearly in the answer box below each question. - You may use anatomical terminology without defining it. - Time will be provided at each pause to complete your response before the video continues. - There are 14 questions in total. - *This is an individual assessment. Do not discuss answers with other students during the session.*   *While watching this surgical video your aim should be to understand the surgery as it proceeds and unfolds in relation to the structures being operated.* |
| --- |

| Anonymisation Code: ___________________________ | Date: ________________ College: _______________ |
| --- | --- |

| **Q1** | **Identify the structure demonstrated in this segment of the video.** |
| --- | --- |

| **Q2** | **Identify the ligament demonstrated in this segment.** |
| --- | --- |

| **Q3** | **Describe the vascular structures contained within this ligament and explain why precise identification and ligation of this structure is important during oophorectomy.** |
| --- | --- |

| **Q4** | **Identify the structure being divided. Describe its anatomical course.** |
| --- | --- |

| **Q5** | **Identify the peritoneal structure being opened. Describe its composition and list the structures it contains.** |
| --- | --- |

| **Q6** | **Identify the pelvic organ visible. Describe its anatomical relationship to the uterus at this stage of the procedure and explain why this relationship is surgically important.** |
| --- | --- |

| **Q7** | **Identify the peritoneal fold being incised. Explain the purpose of this incision.** |
| --- | --- |

| **Q8** | **During this stage of anterior dissection, identify the structure requiring protection and explain the anatomical basis for its vulnerability in this plane.** |
| --- | --- |

| **Q9** | **Identify the anatomical space and describe its boundaries.** |
| --- | --- |

| **Q10** | **If posterior dissection proceeds outside the correct anatomical plane in this region, which adjacent structure is at greatest risk of injury? Explain why.** |
| --- | --- |

| **Q11** | **Identify the vessel being sealed in this segment.** |
| --- | --- |

| **Q12** | **Explain why medial positioning of the surgical instrument during sealing of this vessel is critical for operative safety based on its anatomical relationships.** |
| --- | --- |

| **Q13** | **Identify this ligament complex. Describe its functional role in pelvic support.** |
| --- | --- |

| **Q14** | **During closure and transfixation of the vaginal vault, which named anatomical structures are being incorporated in the apical suture? Explain why their inclusion supports long-term pelvic organ support.** |
| --- | --- |

| \| **Q1** \| **Q2** \| **Q3** \| **Q4** \| **Q5** \| **Q6** \| **Q7** \| **Q8** \| **Q9** \| **Q10** \| **Q11** \| **Q12** \| **Q13** \| **Q14** \| \| --- \| --- \| --- \| --- \| --- \| --- \| --- \| --- \| --- \| --- \| --- \| --- \| --- \| --- \| \| /2 \| /2 \| /2 \| /2 \| /2 \| /2 \| /2 \| /2 \| /2 \| /2 \| /2 \| /2 \| /2 \| /2 \|   **Total: _______ / 28** |
| --- | --- | --- | --- | --- | --- | --- | --- | --- | --- | --- | --- | --- | --- | --- | --- | --- | --- | --- | --- | --- | --- | --- | --- | --- | --- | --- | --- | --- |

| **PART D — Scoring Rubric (Examiner Use Only)** |
| --- |

| **Scoring Key**   \| **2 — Full** \| **1 — Partial** \| **0 — Incorrect / Blank** \| \| --- \| --- \| --- \| \| Correct identification AND anatomically sound explanation where required. All expected elements present. \| Correct identification but incomplete or absent explanation; OR correct explanatory principle without correct structure named. \| Incorrect structure, irrelevant response, or no response. \| |
| --- | --- | --- | --- | --- | --- | --- |

| **Q1 — Ovary** *Identification* | |
| --- | --- |
| **2**  Full | Correctly identifies 'ovary'. |
| **1**  Partial | 'adnexa' — indicates recognition of an adnexal structure without anatomical precision. |
| **0**  None | Incorrect structure (e.g., fibroid, lymph node, 'gland') or blank. |
| **Assessor note:** *Accept 'ovary' in any language equivalent. Do not award partial credit for vague terms such as 'pelvic gland', 'organ', or 'structure' — these do not demonstrate anatomical recognition.* | |

| **Q2 — Infundibulopelvic (IP) Ligament** *Identification* | |
| --- | --- |
| **2**  Full | Names 'infundibulopelvic ligament' OR 'suspensory ligament of the ovary' (correct synonyms). |
| **1**  Partial | Any structural description without the name — e.g., *"the peritoneal fold/ligament running from the ovary to the lateral pelvic side wall"* or *"lateral ovarian ligament."* The student clearly identifies the right structure by its location and course, even if they cannot recall the precise name. |
| **0**  None | Incorrect or blank. |
| **Assessor note:** *Do NOT accept utero-ovarian (ovarian proper) ligament as equivalent — it is a distinct structure medial to the IP ligament. Award 1 at most for that response.* | |

| **Q3 — Vascular Content of IP Ligament & Safety Rationale** *Vascular reasoning* | |
| --- | --- |
| **2**  Full | Names both the ovarian artery and ovarian vein. Provides a clinically relevant explanation: they constitute the lateral ovarian blood supply requiring ligation in BSO, with haemorrhage risk if inadequately controlled. A student who also mentions ureteric proximity as the safety rationale for precise — rather than blanket lateral — ligation demonstrates superior operative awareness and should receive full credit if the vessels are correctly named. |
| **1**  Partial | Names only one of the two vessels (artery or vein) with a correct clinical explanation; OR names both vessels without any explanation of relevance. |
| **0**  None | Incorrect vessels named (e.g., iliac, uterine) or blank. Ureteric proximity alone, without naming the ovarian vessels, scores 0 — the question explicitly asks about vascular structures. |
| **Assessor note:** *The ureteric safety rationale is an excellent additional element and reflects an advanced understanding, but it cannot replace identifying the ovarian vessels. The question has two explicit components: name the vessels, and explain the relevance. Both must be addressed for full credit.* | |

| **Q4 — Round Ligament & Course** *Identification + anatomy* | |
| --- | --- |
| **2**  Full | Names 'round ligament'. Describes the full course: uterine cornu → anterior broad ligament → deep inguinal ring → inguinal canal → labia majora. |
| **1**  Partial | Names round ligament but gives an incomplete course |
| **0**  None | Incorrect structure or blank. |
| **Assessor note:** *Accept 'from uterus to labia' as minimal partial (score 1) only. Award 2 only if the deep inguinal ring or inguinal canal is included.* | |

| **Q5 — Broad Ligament & Composition** *Identification + anatomy* | |
| --- | --- |
| **2**  Full | Names 'broad ligament'. States it is a double layer of peritoneum (or 'peritoneal fold'). Lists at least three of: fallopian tube, uterine/ovarian vessels, round ligament, ovarian ligament, nerves/lymphatics, parametrial connective tissue. |
| **1**  Partial | Names broad ligament but gives no composition; OR describes composition without correctly identifying the structure; OR gives less than 3 contents |
| **0**  None | Incorrect or blank. |
| **Assessor note:** *Reward students who describe it as a peritoneal fold rather than a true ligament — this is anatomically precise. If the student also notes that the anterior leaf is opened to access the retroperitoneum or vesicocervical space, award 2 if remaining criteria are met.* | |

| **Q6 — Bladder & Anatomical Relationship** *Identification + spatial + reasoning* | |
| --- | --- |
| **2**  Full | Identifies the urinary bladder. States it lies anterior to the uterus and cervix/lower uterine segment. Explains surgical importance: the bladder base is closely adherent and must be mobilised inferiorly before colpotomy to avoid cystotomy. |
| **1**  Partial | Identifies the bladder and its anterior position but provides no explanation of surgical importance; OR explains the risk without naming the structure. |
| **0**  None | Incorrect or blank. |
| **Assessor note:** *Three elements are required: identification, spatial description, and surgical reasoning. Identification alone awards 1. Full explanation of the operative safety relevance awards 2.* | |

| **Q7 — Uterovesical Peritoneal Fold & Purpose** *Identification + procedural* | |
| --- | --- |
| **2**  Full | Names 'uterovesical peritoneal fold' or 'vesicouterine peritoneal fold'. States the rationale of allowing safe inferior bladder mobilisation off the cervix. |
| **1**  Partial | Correctly identifies the fold but does not state bladder mobilisation; OR mentions the rationale but does not say the correct name of the fold. |
| **0**  None | Incorrect or blank. |
| **Assessor note:** *Terminology note: both 'uterovesical' and 'vesicouterine' are acceptable. Accept 'anterior peritoneal fold at the bladder-uterine junction'. Do NOT accept 'broad ligament'.* | |

| **Q8 — Structure at Risk During Anterior Dissection** *Structure-at-risk reasoning* | |
| --- | --- |
| **2**  Full | Identifies the urinary bladder as the primary at-risk structure AND explains the anatomical basis: the bladder base is closely adherent to the lower uterine segment and cervix, requiring sharp dissection in the correct vesicocervical plane to avoid cystotomy. |
| **1**  Partial | Identifies the bladder as at-risk but provides no anatomical explanation; OR gives a correct anatomical explanation without naming the structure. |
| **0**  None | Incorrect primary structure (e.g., ureter as sole answer) or blank. |
| **Assessor note:** *The ureter is NOT the primary at-risk structure during anterior dissection. Its danger zones are at the uterine artery and cardinal ligament (Q12 level). If a student mentions both bladder and ureter, award 2 only if bladder is primary. Answers citing only the ureter score 0. This distinction must be marked carefully.* | |

| **Q9 — Pouch of Douglas & Boundaries** *Identification + spatial* | |
| --- | --- |
| **2**  Full | Identifies 'Pouch of Douglas' or 'rectouterine pouch'. States at least two correct boundaries: Anterior = posterior uterus/cervix or just uterus/cervix; Posterior = anterior rectum/rectosigmoid or just rectum/ rectosigmoid; Lateral = uterosacral ligaments. |
| **1**  Partial | Identifies POD but states only one correct boundary; OR identifies all correct boundaries without naming the structure. |
| **0**  None | Incorrect or blank. |
| **Assessor note:** *If all three boundaries are correct but the structure is not named, award 1. Both correct identification and at least two boundaries are required for full credit.* | |

| **Q10 — At-Risk Structure Posteriorly** *Structure-at-risk reasoning* | |
| --- | --- |
| **2**  Full | Identifies the rectum (or rectosigmoid) AND explains why: it forms the posterior boundary of the POD, and dissection outside the correct posterior plane risks serosal or full-thickness rectal injury. |
| **1**  Partial | 'Bowel' without specifying the rectum, with a correct explanation of its posterior boundary role OR Identifies rectum but does not explain why OR does not identify structure as rectum but explains why |
| **0**  None | Incorrect structure (e.g., ureter, sacrum) or blank. |
| **Assessor note:** *Ureter-only answers are incorrect in the context of this question (posterior peritoneal entry). Students who conflate the rectal danger zone with the ureteric danger zone show incomplete spatial understanding.* | |

| **Q11 — Uterine Artery** *Identification* | |
| --- | --- |
| **2**  Full | Identifies 'uterine artery'. |
| **1**  Partial | 'artery to the uterus', or 'branch of the Internal Iliac Artery', without specifically naming it the uterine artery. |
| **0**  None | Incorrect vessel (e.g., ovarian artery at this stage) or blank. |
| **Assessor note:** *Pure identification item. Award 2 for 'uterine artery' only.* | |

| **Q12 — Uterine Artery Relationships & Medial Positioning** *Relational + reasoning* | |
| --- | --- |
| **2**  Full | Explains that medial instrument positioning protects the ureter (e.g., 'to avoid the ureter that passes immediately inferior to the uterine artery', or 'to avoid the ureter, which passes beneath the artery here'). |
| **1**  Partial | Explains medial positioning rationale to avoid injury (to organ/ structure etc) without mentioning ureter OR mentions ureter but does not explicitly specify relation between ureter and the artery. |
| **0**  None | Incorrect or anatomically unrelated reasoning. |
| **Assessor note:** *Assessor key: the ureter passes immediately INFERIOR to (beneath) the uterine artery within the parametrium — the classic description is 'water under the bridge'. Students who correctly name the ureter and describe it passing inferior to the artery receive full credit. Students who correctly name the ureter and describe its lateral location to uterus, also receive full credit* | |

| **Q13 — Cardinal (Mackenrodt's) Ligament** *Identification + function* | |
| --- | --- |
| **2**  Full | Names 'cardinal ligament', 'Mackenrodt's ligament', or 'transverse cervical ligament', or ‘lateral cervical ligament’ and/o uterosacral ligament. Describes relevance as primary pelvic support. |
| **1**  Partial | Correctly identifies the ligament(s) but does not address relevance OR identifies relevance, but does not name the ligament(s) |
| **0**  None | Incorrect or blank. |
| **Assessor note:** *Award 2 for any anatomically sound answer identifying either or both of these ligaments* | |

| **Q14 — Vaginal Cuff Transfixation & Apical Support** *Procedural integration* | |
| --- | --- |
| **2**  Full | Names at least the uterosacral ligaments OR/AND cardinal ligaments as structures classically incorporated in the apical suture. Explains that their inclusion reconstructs apical pelvic support and reduces the risk of post-hysterectomy vault prolapse. |
| **1**  Partial | Names uterosacral and/or cardinal ligament but without an explanation OR does not name either ligament but explains the rationale of some ligamentous/ support structure being attached to prevent vault prolapse. |
| **0**  None | Incorrect structures (e.g., broad ligament, round ligament) or blank. |
| **Assessor note:** *Award 2 for any anatomically sound answer identifying either or both of these ligaments. 'Parametrium' alone without naming specific structures scores 0.* | |

| **Scoring Summary**  Total Score = sum of all 14 items (range 0–28). Higher scores indicate greater surgical anatomical understanding.   \| **Domain** \| **Items (scored 0–2 each)** \| \| --- \| --- \| \| Identification (pure recall) \| Q1, Q2, Q11 \| \| Identification + Applied Anatomy Knowledge \| Q4, Q5, Q7, Q9, Q13 \| \| Relational / Spatial \| Q3, Q5, Q6, Q7, Q12 \| \| Structure-at-Risk Reasoning \| Q8, Q10 \| \| Procedural Integration \| Q14 \| |
| --- | --- | --- | --- | --- | --- | --- | --- | --- | --- | --- | --- | --- |

| **PART E — Construct-to-Question Mapping Matrix** |
| --- |

This matrix shows the anatomical constructs from Part A that are directly and fully assessed by the 14-item instrument. Only constructs with a direct question item are listed.

| **Q** | **Construct Assessed** | **Domain / Structure Type** | Contextual Construct Tested |
| --- | --- | --- | --- |
| **Q1** | Ovary | *Visceral* | Identification |
| **Q2** | Infundibulopelvic (suspensory) ligament | *Ligamentous / Support* | Identification |
| **Q3** | Ovarian artery | *Vascular* | Relational / Spatial |
| **Q3** | Ovarian vein | *Vascular* | Relational / Spatial |
| **Q4** | Round ligament | *Ligamentous / Support* | Identification + Applied Anatomy Knowledge |
| **Q5** | Broad ligament | *Peritoneal* | Relational / Spatial |
| **Q6** | Urinary bladder | *Visceral* | Relational / Spatial |
| **Q7** | Uterovesical peritoneal fold | *Peritoneal* | Identification + Applied Anatomy Knowledge |
| **Q8** | Urinary bladder | *Visceral* | Structure-at-risk reasoning |
| **Q9** | Pouch of Douglas (rectouterine pouch) | *Peritoneal* | Identification + Applied Anatomy Knowledge |
| **Q10** | Rectum / rectosigmoid | *Visceral* | Identification + Applied Anatomy Knowledge |
| **Q11** | Uterine artery | *Vascular* | Identification |
| **Q12** | Ureter | *Urinary Tract* | Relational / Spatial |
| **Q13** | Cardinal (Mackenrodt's) ligament | *Ligamentous / Support* | Identification + Applied Anatomy Knowledge |
| **Q13** | Uterosacral ligament | *Ligamentous / Support* | Identification + Applied Anatomy Knowledge |
| **Q13** | Cardinal and Uterosacral ligaments | *Ligamentous / Support* | Identification + Applied Anatomy Knowledge |
| **Q14** | Vagina / vaginal vault | *Visceral* | Procedural integration |
| **Q14** | Uterosacral ligaments | *Ligamentous / Support* | Procedural integration |
| **Q14** | Cardinal (Mackenrodt's) ligament | *Ligamentous / Support* | Procedural integration |
