# Supplementary material for: The instructional modality used for contextual vertical integration of anatomy influences cognitive load and performance during an operative interpretation task in undergraduate gynaecology students: evidence from a multi-centre cluster-randomised controlled trial
Source: Front Med (Lausanne). 2026 May 21;13:1827733. doi: 10.3389/fmed.2026.1827733 (PMC13250826; doi:10.3389/fmed.2026.1827733)
Supplement: Supplementary material 2 — Adapted operative-interpretation cognitive load questionnaire. [file Supplementary_file_2.docx]

**Supplementary Material 2**

**Cognitive Load during Operative Interpretation Task (CL_OI_) Questionnaire**

Adapted from Krieglstein, F., Beege, M., Rey, G. D., Sanchez-Stockhammer, C., & Schneider, S. (2023). Development and Validation of a Theory-Based Questionnaire to Measure Different Types of Cognitive Load. *Educational Psychology Review 2023 35:1*, *35*(1), 1–37. https://doi.org/10.1007/S10648-023-09738-0

|  |
| --- |

| **Instructions to Students**  You have just watched a video of a Total Laparoscopic Hysterectomy with Bilateral Salpingo-Oophorectomy (TLH+BSO). Please answer the questions below based on your experience of watching the procedure.  For each statement, circle the number that best reflects your experience.  **1 = Not at all applicable 9 = Fully applicable**  *There are no right or wrong answers. Please respond to every item.* |
| --- |

| Anonymisation Code: ___________________________ | Date: ________________ College: _______________ |
| --- | --- |

| **1** | The operative procedure was difficult to understand. |
| --- | --- |
|  | \| **1** \| 2 \| 3 \| 4 \| 5 \| 6 \| 7 \| 8 \| **9** \| \| --- \| --- \| --- \| --- \| --- \| --- \| --- \| --- \| --- \| \| *Not at all*  *applicable* \|  \|  \|  \|  \|  \|  \|  \| *Fully*  *applicable* \| |

| **2** | The explanation of the operative procedure was difficult to understand. |
| --- | --- |
|  | \| **1** \| 2 \| 3 \| 4 \| 5 \| 6 \| 7 \| 8 \| **9** \| \| --- \| --- \| --- \| --- \| --- \| --- \| --- \| --- \| --- \| \| *Not at all*  *applicable* \|  \|  \|  \|  \|  \|  \|  \| *Fully*  *applicable* \| |

| **3** | The operative procedure was complex. |
| --- | --- |
|  | \| **1** \| 2 \| 3 \| 4 \| 5 \| 6 \| 7 \| 8 \| **9** \| \| --- \| --- \| --- \| --- \| --- \| --- \| --- \| --- \| --- \| \| *Not at all*  *applicable* \|  \|  \|  \|  \|  \|  \|  \| *Fully*  *applicable* \| |

| **4** | The operative procedure included many complex steps. |
| --- | --- |
|  | \| **1** \| 2 \| 3 \| 4 \| 5 \| 6 \| 7 \| 8 \| **9** \| \| --- \| --- \| --- \| --- \| --- \| --- \| --- \| --- \| --- \| \| *Not at all*  *applicable* \|  \|  \|  \|  \|  \|  \|  \| *Fully*  *applicable* \| |

| **5** | Without prior knowledge, the operative procedure would not have been understandable. |
| --- | --- |
|  | \| **1** \| 2 \| 3 \| 4 \| 5 \| 6 \| 7 \| 8 \| **9** \| \| --- \| --- \| --- \| --- \| --- \| --- \| --- \| --- \| --- \| \| *Not at all*  *applicable* \|  \|  \|  \|  \|  \|  \|  \| *Fully*  *applicable* \| |

| **6** | It was difficult to gain an overview of the operative procedure as presented. |
| --- | --- |
|  | \| **1** \| 2 \| 3 \| 4 \| 5 \| 6 \| 7 \| 8 \| **9** \| \| --- \| --- \| --- \| --- \| --- \| --- \| --- \| --- \| --- \| \| *Not at all*  *applicable* \|  \|  \|  \|  \|  \|  \|  \| *Fully*  *applicable* \| |

| **7** | The way the operative procedure was presented made it difficult to recognise links between individual steps and relevant anatomical structures. |
| --- | --- |
|  | \| **1** \| 2 \| 3 \| 4 \| 5 \| 6 \| 7 \| 8 \| **9** \| \| --- \| --- \| --- \| --- \| --- \| --- \| --- \| --- \| --- \| \| *Not at all*  *applicable* \|  \|  \|  \|  \|  \|  \|  \| *Fully*  *applicable* \| |

| **8** | The presentation of the operative procedure was inconvenient for learning. |
| --- | --- |
|  | \| **1** \| 2 \| 3 \| 4 \| 5 \| 6 \| 7 \| 8 \| **9** \| \| --- \| --- \| --- \| --- \| --- \| --- \| --- \| --- \| --- \| \| *Not at all*  *applicable* \|  \|  \|  \|  \|  \|  \|  \| *Fully*  *applicable* \| |

| **9** | The presentation of the operative procedure made it difficult to quickly identify relevant anatomical structures. |
| --- | --- |
|  | \| **1** \| 2 \| 3 \| 4 \| 5 \| 6 \| 7 \| 8 \| **9** \| \| --- \| --- \| --- \| --- \| --- \| --- \| --- \| --- \| --- \| \| *Not at all*  *applicable* \|  \|  \|  \|  \|  \|  \|  \| *Fully*  *applicable* \| |

| **10** | Because of the way the operative procedure was presented, I had the impression that I could not concentrate on the key steps and relevant anatomical structures. |
| --- | --- |
|  | \| **1** \| 2 \| 3 \| 4 \| 5 \| 6 \| 7 \| 8 \| **9** \| \| --- \| --- \| --- \| --- \| --- \| --- \| --- \| --- \| --- \| \| *Not at all*  *applicable* \|  \|  \|  \|  \|  \|  \|  \| *Fully*  *applicable* \| |

| **11** | I actively reflected upon the steps of the operative procedure and the relevant anatomical structures. |
| --- | --- |
|  | \| **1** \| 2 \| 3 \| 4 \| 5 \| 6 \| 7 \| 8 \| **9** \| \| --- \| --- \| --- \| --- \| --- \| --- \| --- \| --- \| --- \| \| *Not at all*  *applicable* \|  \|  \|  \|  \|  \|  \|  \| *Fully*  *applicable* \| |

| **12** | I made an effort to understand the operative procedure and the relevant anatomical structures. |
| --- | --- |
|  | \| **1** \| 2 \| 3 \| 4 \| 5 \| 6 \| 7 \| 8 \| **9** \| \| --- \| --- \| --- \| --- \| --- \| --- \| --- \| --- \| --- \| \| *Not at all*  *applicable* \|  \|  \|  \|  \|  \|  \|  \| *Fully*  *applicable* \| |

| **13** | I achieved a comprehensive understanding of the operative procedure and the relevant anatomical structures. |
| --- | --- |
|  | \| **1** \| 2 \| 3 \| 4 \| 5 \| 6 \| 7 \| 8 \| **9** \| \| --- \| --- \| --- \| --- \| --- \| --- \| --- \| --- \| --- \| \| *Not at all*  *applicable* \|  \|  \|  \|  \|  \|  \|  \| *Fully*  *applicable* \| |

| **14** | I was able to expand my prior knowledge through observing the operative procedure and the relevant anatomical structures. |
| --- | --- |
|  | \| **1** \| 2 \| 3 \| 4 \| 5 \| 6 \| 7 \| 8 \| **9** \| \| --- \| --- \| --- \| --- \| --- \| --- \| --- \| --- \| --- \| \| *Not at all*  *applicable* \|  \|  \|  \|  \|  \|  \|  \| *Fully*  *applicable* \| |

| **15** | I can apply the knowledge that I acquired in this session quickly and accurately. |
| --- | --- |
|  | \| **1** \| 2 \| 3 \| 4 \| 5 \| 6 \| 7 \| 8 \| **9** \| \| --- \| --- \| --- \| --- \| --- \| --- \| --- \| --- \| --- \| \| *Not at all*  *applicable* \|  \|  \|  \|  \|  \|  \|  \| *Fully*  *applicable* \| |

| Please rate the **mental effort** you invested while observing and trying to understand the laparoscopic hysterectomy procedure and relevant anatomy in the video you have just watched. Circle one number. |
| --- |

| **1** | Very, very low mental effort |
| --- | --- |
| **2** | Very low mental effort |
| **3** | Low mental effort |
| **4** | Rather low mental effort |
| **5** | Neither low nor high mental effort |
| **6** | Rather high mental effort |
| **7** | High mental effort |
| **8** | Very high mental effort |
| **9** | Very, very high mental effort |
