# Supplementary material for: The instructional modality used for contextual vertical integration of anatomy influences cognitive load and performance during an operative interpretation task in undergraduate gynaecology students: evidence from a multi-centre cluster-randomised controlled trial
Source: Front Med (Lausanne). 2026 May 21;13:1827733. doi: 10.3389/fmed.2026.1827733 (PMC13250826; doi:10.3389/fmed.2026.1827733)
Supplement: Supplementary material 3 — Costing assumptions and calculations for the cost–consequence analysis. [file Supplementary_file_3.docx]

**Supplementary Material 3**

**Cost–Consequence Analysis**

*Detailed Methodology, Assumptions, and Results*

| **Sections**  1. Analytic Framework  2. Cost Identification: Included and Excluded Items  3. Unit Costs and Data Sources  4. Denominator and Linkage to Outcomes  5. Excluded Research-Specific Costs  6. Detailed Results (Tables S1, S2)  7. Sensitivity, Scalability, and Transferability  8. Conflicts of Interest Relevant to Cost Analysis |
| --- |

| **1** | **Analytic Framework** |
| --- | --- |

**1.1 Type of Economic Evaluation**

A cost–consequence analysis (CCA) was selected rather than a cost-effectiveness analysis (CEA). In a CCA, costs and outcomes are presented in parallel in a disaggregated format, without combining them into a single ratio. This was appropriate for three reasons: (a) the study measured multiple non-commensurate outcomes (three cognitive load subscales and a surgical anatomy performance score), none of which can be straightforwardly combined into a single effectiveness metric; (b) decision-makers in medical education settings typically weigh multiple dimensions simultaneously (learning outcomes, feasibility, institutional capacity) rather than optimising on a single ratio; and (c) CCA provides maximum transparency, allowing stakeholders to apply their own value judgements to the trade-offs between cost and outcomes.

**1.2 Perspective**

The analysis adopted the institutional perspective, capturing only direct costs borne by the medical college in delivering each instructional modality. This is the most relevant perspective for curriculum decision-makers within medical institutions. Student time costs, opportunity costs, and broader societal costs were excluded, as they did not differ across modalities — all sessions were of identical duration and delivered within scheduled curricular time.

**1.3 Time Horizon and Discounting**

The time horizon was the study delivery period within a single academic year (December 2025–January 2026). No extrapolation beyond the observed data was undertaken. As the time horizon was one year or less, discounting was not applied, consistent with standard health economic practice.

**1.4 Scope**

This was a trial-based economic evaluation conducted alongside the cluster-randomised controlled trial. No decision-analytic modelling was employed. All cost and outcome data were drawn directly from the trial. Because the contextually integrated anatomy teaching programme described in this study did not exist prior to the trial, costs reflect the actual resources consumed in delivering the programme to this cohort (11 sessions per arm, approximately 130 students per arm), rather than a projection onto a hypothetical annual programme. Implications for cost at scale are discussed in Section 7.

| **2** | **Cost Identification: Included and Excluded Items** |
| --- | --- |

The analysis focused on costs relevant to modality choice. Faculty personnel costs were included in full because overall faculty costs varied across modalities owing to differences in preparation requirements, even though some faculty components were identical across arms. Common non-personnel costs, including teaching rooms and shared equipment, were excluded because they did not vary by modality and therefore did not affect comparative interpretation. The table below documents each cost item, the inclusion or exclusion decision, and the rationale.

| **Item** | **Decision and Value** | **Rationale** |
| --- | --- | --- |
| Faculty preparation time | Included: differs by modality | Preparation requirements differed across modalities (prosection preparation, software familiarisation, slide creation) and represent a genuine differential cost. |
| Faculty teaching time | Included: identical across arms (₹13,200 per arm) | Included as part of total faculty personnel costs, which were retained in full because overall faculty costs varied by modality owing to preparation requirements. |
| Technician time | Included | Technician involvement in cadaveric prosection (CP) preparation and session facilitation was specific to the cadaveric arm and absent in the interactive three-dimensional digital (I3DD) and slide-based (SB) arms. |
| Cadaver procurement | Included (apportioned) | Direct material cost specific to CP arm; apportioned by dissection region (see Section 3.4). |
| Cadaver maintenance | Included (apportioned) | Recurring annual institutional cost; apportioned per cadaver and by region. |
| Embalming chemicals | Included (apportioned) | Recurring annual consumable cost; apportioned per cadaver and by region. |
| PPE (CP only) | Included: ₹900/session | Consumable cost (gloves, gowns) specific to cadaveric sessions. |
| 3D Organon software licence | Included | Annual licence specific to the I3DD arm; the only differential technology cost. |
| Teaching rooms | Excluded | Identical across all three arms; institutionally owned at no marginal cost. |
| Projector / display screen | Excluded | Shared equipment used across all arms. |
| Presentation software (SB) | Excluded | Institutionally licensed software already in routine use. |
| Tablet hardware (3D) | Excluded | Existing institutional equipment used across all arms; not purchased for the study. |
| Student time | Excluded | Session duration identical across all arms (60 min); no differential opportunity cost. |
| Cadaver transport across institutions | Excluded (research-specific) | Incurred solely to standardise the prosection for the trial. In routine implementation, each college would use its own cadavers (see Section 5). |
| Assessment and data collection | Excluded (research-specific) | Identical across arms; would not be incurred in routine curricular delivery. |

| **3** | **Unit Costs and Data Sources** |
| --- | --- |

**3.1 Personnel Costs**

| **Item** | **Decision and Value** | **Rationale** |
| --- | --- | --- |
| Faculty hourly rate | ₹1,200 per hour | Based on the salary scale of the faculty member who delivered all teaching sessions across all three institutions and all three arms. |
| Technician hourly rate | ₹300 per hour | Based on the salary scale of anatomy department technicians at the participating institutions. |

**3.2 Faculty Preparation Time by Modality**

| **Item** | **Decision and Value** | **Rationale** |
| --- | --- | --- |
| CP: Prosection preparation | 8 hours (one-off) | Time for the anatomist to prepare the abdominopelvic prosection, including retroperitoneal dissection of the left ureter and preservation of the contralateral broad ligament. One-off; the same specimen was used for all 11 CP sessions. |
| CP: Delphi mapping and teaching design | 2 hours (one-off) | Time to map Delphi-derived anatomical constructs onto the prosection and design the standardised lesson plan. |
| CP: Per-session preparation | Nil | No additional preparation required for individual sessions. |
| I3DD: Software familiarisation | 3 hours (one-off) | Time for the instructor to become proficient with the 3D Organon interface (rotation, layering, selective display, procedural sequencing). |
| I3DD: Delphi mapping and teaching design | 2 hours (one-off) | As above for CP. |
| SB: Slide preparation | 3 hours (one-off) | Time to source, annotate, and sequence high-resolution anatomical images into a standardised slide presentation. |
| SB: Delphi mapping and teaching design | 2 hours (one-off) | As above for CP and I3DD. |

**3.3 Technician Time (CP Arm Only)**

| **Item** | **Decision and Value** | **Rationale** |
| --- | --- | --- |
| Prosection preparation | 2 technicians × 6 hours = 12 person-hours (one-off) | Two technicians assisted in preparing the prosection specimen prior to any teaching sessions. |
| Session facilitation | 1 technician × 1 hour × 11 sessions = 11 person-hours | A technician remained present throughout each CP session to facilitate specimen handling and assist the instructor. Not required in I3DD or SB arms. |

**3.4 Cadaver Costs and Apportionment**

**Raw institutional cost data.** Cadaver procurement: ₹1,30,000 per cadaver. Annual maintenance: ₹6,00,000 for 15 cadavers (₹40,000 per cadaver per annum). Annual embalming chemicals: ₹4,50,000 for 15 cadavers (₹30,000 per cadaver per annum). All figures were provided by the anatomy departments of the participating institutions and reflect 2025–26 prices. One cadaver was used across the study.

**Apportionment logic.** A single cadaver is used across the full anatomy curriculum for multiple dissection regions. To attribute an appropriate share of cadaver costs to this study's abdominopelvic prosection, costs were apportioned equally across the five standard anatomical dissection regions used in the undergraduate medical curriculum: (1) Upper limb, (2) Lower limb, (3) Thorax, (4) Abdomen & pelvis, (5) Head & neck. The abdominopelvic prosection falls within a single region (region 4), yielding an apportionment fraction of one-fifth (20%) of per-cadaver costs.

**Choice of apportionment fraction.** The one-fifth fraction was deliberately selected as the most conservative (lowest) estimate of cadaver costs attributable to this study, ensuring any cost advantage observed for the I3DD arm is robust and not an artefact of inflated cadaveric costs. Three apportionment approaches are presented:

| **Item** | **Decision and Value** | **Rationale** |
| --- | --- | --- |
| **1/5 (20%) — PRIMARY ANALYSIS** | Abdomen & pelvis as a single region among five standard dissection regions | Most conservative estimate; minimises cadaver costs. Aligned with the standard organisation of major dissection manuals (e.g., Cunningham's Manual). Provides the most stringent test of the I3DD arm's cost advantage. |
| 1/3 (33%) — alternative | Abdomen and pelvis as two separate regions among six | Some anatomy curricula enumerate abdomen and pelvis as distinct dissection units. Would increase CP per-student cost to approximately ₹823. |
| 28% — alternative | Body-segment weight proportions | Empirically derived from anthropometric data (Winter 1990; Dempster 1955). Would yield CP per-student cost of approximately ₹742. Less directly relevant to teaching utilisation than region-based apportionment. |

| Under all three apportionment assumptions, the I3DD per-student cost (₹361) remains substantially below CP, confirming that the conclusion is robust to the apportionment method used. |
| --- |

**3.5 PPE Costs (CP Arm Only)**

Personal protective equipment (gloves, gowns) was required for students and the instructor during each cadaveric session. The cost of ₹900 per session was based on actual institutional procurement records. PPE was not required for I3DD or SB sessions. Total: ₹900 × 11 sessions = ₹9,900.

**3.6 Software Licence (I3DD Arm Only)**

The 3D Organon annual licence cost was USD 299.50, converted to INR at the prevailing exchange rate during the study period (December 2025–January 2026) of approximately ₹90 per USD, yielding **₹26,955**.

| **Research licence disclosure.** The 3D Organon software licence used in this study was provided for research purposes. The manufacturer had no role in study design, data collection, analysis, interpretation, or manuscript preparation. The cost–consequence analysis costed the licence at the standard annual institutional rate (USD 299.50), which is the price an adopting institution would pay. This ensures the cost analysis reflects real-world implementation costs rather than the research-specific procurement arrangement.  **Symmetry of resource sharing across institutions.** A single software licence was applied across all three institutions, paralleling the use of a single cadaveric prosection across all three institutions. Both decisions were research-specific measures to standardise instructional delivery across sites. In routine curricular implementation, each institution would be expected to procure its own 3D Organon licence, just as each institution would utilise its own cadaveric resources. The relative cost advantage of I3DD over CP would be expected to persist, as each institution's cadaver procurement, maintenance, and consumable costs would similarly apply. |
| --- |

| **4** | **Denominator and Linkage to Outcomes** |
| --- | --- |

**4.1 Per-Student Cost Denominator**

Costs were incurred for all 400 students who attended the scheduled teaching sessions, irrespective of research consent. Of these, 394 provided written consent for research data collection. Per-student costs were calculated using the **consented-and-taught sample** (CP = 132, I3DD = 128, SB = 134) as the denominator, as these are the students for whom both costs and at least some outcome data are available.

**4.2 Outcome Sample Sizes**

| **Item** | **Decision and Value** | **Rationale** |
| --- | --- | --- |
| Anatomy teaching CL (secondary) | n = 394 (CP = 132, I3DD = 128, SB = 134) | Measured immediately post-instruction; available for all consenting participants. |
| Operative interpretation CL (primary) | n = 383 (CP = 128, I3DD = 125, SB = 130) | Measured at one-week follow-up; 11 consenting students did not attend the assessment session. |
| Surgical anatomy score (primary) | n = 383 (CP = 128, I3DD = 125, SB = 130) | Same assessment session as operative interpretation CL. |

*The slight discrepancy between the cost denominator (n = 394) and the primary outcome denominator (n = 383) is inherent to the cost–consequence design: costs are incurred at the point of teaching delivery, whereas outcomes are measured subsequently. The cost–consequence format presents these in parallel with explicit footnotes, avoiding the need to force alignment into a single ratio.*

| **5** | **Excluded Research-Specific Costs** |
| --- | --- |

**Cadaver Transport Across Institutions**

A single cadaveric prosection was prepared and transported across all three participating colleges to ensure standardisation. This transport cost was a research-specific expenditure incurred solely to maintain internal validity. In routine implementation, each institution would use its own cadaveric resources. Including this cost would overestimate the true institutional cost of cadaveric teaching and reduce external validity. The exclusion is noted in the main manuscript.

**Assessment and Data Collection**

Costs associated with outcome assessment (video preparation, questionnaire printing, script scoring) were identical across all three arms and research specific. They were excluded because they do not represent costs incurred in routine delivery.

**Ethical Parity Sessions**

Optional drop-in sessions offering access to all three modalities were provided after study completion to ensure ethical parity. These occurred after data collection and did not contribute to study outcomes or costs.

| **6** | **Detailed Results** |
| --- | --- |

**6.1 Calculation Trace**

The following table provides the complete arithmetic for every cost line, enabling independent verification of all reported figures.

| **Item** | **Decision and Value** | **Rationale** |
| --- | --- | --- |
| CP faculty | (8 + 2) × ₹1,200 + (11 × ₹1,200) = ₹12,000 + ₹13,200 = ₹25,200 | Prosection prep (8 hrs) + Delphi (2 hrs) + 11 teaching sessions (11 hrs). All at ₹1,200/hr. |
| CP technician | (12 × ₹300) + (11 × ₹300) = ₹3,600 + ₹3,300 = ₹6,900 | Prosection prep (2 techs × 6 hrs) + session facilitation (11 × 1 hr). All at ₹300/hr. |
| CP cadaver procurement | ₹1,30,000 × 1/5 = ₹26,000 | One cadaver, apportioned at 1/5 for abdominopelvic region. |
| CP cadaver maintenance | (₹6,00,000 ÷ 15) × 1/5 = ₹40,000 × 0.2 = ₹8,000 | Annual maintenance per cadaver (₹40,000), apportioned at 1/5. |
| CP embalming chemicals | (₹4,50,000 ÷ 15) × 1/5 = ₹30,000 × 0.2 = ₹6,000 | Annual embalming per cadaver (₹30,000), apportioned at 1/5. |
| CP PPE | ₹900 × 11 = ₹9,900 | Per-session PPE × number of sessions. |
| **CP TOTAL** | ₹25,200 + ₹6,900 + ₹26,000 + ₹8,000 + ₹6,000 + ₹9,900 = ₹82,000 | Sum of all CP-specific costs. |
| **CP per student** | ₹82,000 ÷ 132 = ₹621 | Denominator: consented students who received CP teaching. |
| I3DD faculty | (3 + 2) × ₹1,200 + (11 × ₹1,200) = ₹6,000 + ₹13,200 = ₹19,200 | Software familiarisation (3 hrs) + Delphi (2 hrs) + 11 sessions. |
| I3DD software licence | USD 299.50 × ₹90 = ₹26,955 | Annual licence at study-period exchange rate. |
| **I3DD TOTAL** | ₹19,200 + ₹26,955 = ₹46,155 | Faculty + software. |
| **I3DD per student** | ₹46,155 ÷ 128 = ₹361 | Denominator: consented students who received 3D teaching. |
| SB faculty | (3 + 2) × ₹1,200 + (11 × ₹1,200) = ₹6,000 + ₹13,200 = ₹19,200 | Slide preparation (3 hrs) + Delphi (2 hrs) + 11 sessions. |
| **SB TOTAL** | ₹19,200 | Faculty cost only; no other differential costs. |
| **SB per student** | ₹19,200 ÷ 134 = ₹143 | Denominator: consented students who received SB teaching. |

**6.2 Itemised Cost Breakdown (Table S1)**

Table S1 presents the full itemised cost breakdown by instructional modality.

**Table S1.** *Itemised cost breakdown by instructional modality (2025–26 INR, institutional perspective)*

| **Cost Component** | **CP** | **I3DD** | **SB** |
| --- | --- | --- | --- |
| **Faculty costs** | | | |
| Modality-specific preparation | ₹9,600 | ₹3,600 | ₹3,600 |
| Delphi mapping and teaching design | ₹2,400 | ₹2,400 | ₹2,400 |
| Teaching delivery (11 sessions × 1 hr) | ₹13,200 | ₹13,200 | ₹13,200 |
| **Faculty subtotal** | **₹25,200** | **₹19,200** | **₹19,200** |
| **Technician costs (CP only)** | | | |
| Prosection preparation (2 × 6 hrs) | ₹3,600 | — | — |
| Session facilitation (11 × 1 hr) | ₹3,300 | — | — |
| **Technician subtotal** | **₹6,900** | **—** | **—** |
| **Cadaveric material costs (CP only)** | | | |
| Cadaver procurement (1/5 apportioned) | ₹26,000 | — | — |
| Annual maintenance (1/5 apportioned) | ₹8,000 | — | — |
| Annual embalming (1/5 apportioned) | ₹6,000 | — | — |
| **Cadaveric subtotal** | **₹40,000** | **—** | **—** |
| **Consumables and technology** | | | |
| PPE (CP — 11 sessions × ₹900) | ₹9,900 | — | — |
| 3D Organon annual licence (USD 299.50 × ₹90) | — | ₹26,955 | — |
| **Total programme cost** | **₹82,000** | **₹46,155** | **₹19,200** |
| **Cost per consented-and-taught studentᵃ** | **₹621 (n=132)** | **₹361 (n=128)** | **₹143 (n=134)** |

*Cadaver costs apportioned at 1/5 (abdomen & pelvis as a single region among five standard dissection regions). Faculty rate: ₹1,200/hr; technician rate: ₹300/hr. USD–INR exchange rate: ₹90 (December 2025–January 2026). Common non-personnel costs shared across arms (teaching rooms, shared equipment) were excluded. Faculty personnel costs were retained in full because overall faculty costs varied across modalities owing to differences in preparation requirements.*

**6.3 Cost–Consequence Summary (Table S2)**

Table S2 presents institutional costs alongside all primary and secondary learning outcomes, allowing decision-makers to weigh resource implications against effectiveness without aggregation into a single ratio.

**Table S2.** *Cost–consequence summary: institutional costs alongside learning outcomes by instructional modality*

| **Outcome** | **CP** | **I3DD** | **SB** |
| --- | --- | --- | --- |
| **Costs (institutional perspective, 2025–26 INR)** | | | |
| Total programme cost | **₹82,000** | **₹46,155** | **₹19,200** |
| Cost per consented studentᵃ | **₹621** | **₹361** | **₹143** |
| **Primary outcomes: Operative interpretation cognitive load, EMM (SE)ᵇ** | | | |
| ICL_OI_ | 28.70 (0.38) | 27.64 (0.39) | 32.68 (0.38) |
| ECL_OI_ | 26.53 (0.42) | 26.41 (0.43) | 34.75 (0.42) |
| GCL_OI_ | 37.45 (0.47) | 38.28 (0.47) | 28.88 (0.46) |
| **Primary outcome: Surgical anatomy performance, EMM (SE)ᵇ** | | | |
| Surgical anatomy score (/28) | 21.96 (0.42) | 22.08 (0.42) | 18.44 (0.41) |
| **Secondary outcomes: Anatomy teaching cognitive load, EMM (SE)ᶜ** | | | |
| ICL_AT_ | 32.37 (0.39) | 29.59 (0.40) | 34.42 (0.39) |
| ECL_AT_ | 33.23 (0.41) | 31.01 (0.42) | 35.56 (0.41) |
| GCL_AT_ | 38.33 (0.46) | 37.97 (0.47) | 31.87 (0.46) |

*CP = cadaveric prosection; I3DD = interactive three-dimensional anatomy; SB = slide-based instruction; EMM = estimated marginal mean; SE = standard error; ICL = intrinsic cognitive load; ECL = extraneous cognitive load; GCL = germane cognitive load; OI = operative interpretation; AT = anatomy teaching. Cognitive load subscales scored 5–45; higher ICL/ECL = greater cognitive burden; higher GCL = greater productive processing. Surgical anatomy scored 0–28. ᵃ Per-student costs calculated using consented-and-taught student (CP = 132, I3DD = 128, SB = 134). ᵇ Primary outcomes assessed at one-week follow-up (n = 383: CP = 128, I3DD = 125, SB = 130). ᶜ Secondary outcomes assessed immediately post-instruction (n = 394: CP = 132, I3DD = 128, SB = 134).*

| **7** | **Sensitivity, Scalability, and Transferability** |
| --- | --- |

**7.1 Apportionment Sensitivity**

Table S3 presents per-student CP costs under all three apportionment assumptions, demonstrating that the I3DD cost advantage is robust.

**Table S3.** *Sensitivity of CP per-student cost to cadaver apportionment assumption*

| **Item** | **Decision and Value** | **Rationale** |
| --- | --- | --- |
| **1/5 (20%) — primary analysis** | CP per student = ₹621 | Most conservative; minimises CP costs. |
| 28% (body weight) | CP per student ≈ ₹742 | Intermediate estimate. |
| 1/3 (33%) | CP per student ≈ ₹823 | Highest estimate; maximises CP costs. |
| I3DD per student (unchanged) | ₹361 | Not affected by cadaver apportionment. |
| SB per student (unchanged) | ₹143 | Not affected by cadaver apportionment. |

| Under all scenarios, I3DD remains substantially less expensive than CP, while no statistically significant difference in learning outcomes was observed between the two modalities in the superiority analysis. |
| --- |

**7.2 Scalability**

Per-student costs are sensitive to cohort size because several cost components are fixed (one-off preparation, cadaver procurement, software licence) while others are variable per session (teaching time, technician facilitation, PPE). At larger cohort sizes and more sessions, fixed costs are spread across more students, reducing per-student costs across all modalities. However, the variable costs specific to CP (technician time per session, PPE per session) mean that the CP–I3DD cost gap would persist or widen at scale. Conversely, if only a single session were delivered, the fixed software licence cost for the I3DD arm would weigh more heavily per student.

| **Institutional adoption note.** In routine curricular implementation, each institution would be expected to procure its own cadaveric resources and its own 3D Organon software licence. Because the study applied both a single cadaveric prosection and a single software licence across all three institutions, the costing treats these symmetrically. The software licence (₹26,955 per annum) remains a fraction of the per-institution cadaveric costs (procurement, annual maintenance, embalming, technician time, and PPE), preserving the relative cost advantage of the I3DD modality. |
| --- |

**7.3 Transferability**

Unit costs (faculty rates, technician rates, cadaver procurement) reflect the participating institutions in 2025–26 (see main manuscript for setting description). The software licence is priced in USD and may vary depending on the institutional subscription type. Cadaver availability and cost structures differ substantially across countries and institutions, particularly between well-resourced and resource-constrained settings. Decision-makers in other contexts should substitute local unit costs.

| **8** | **Conflicts of Interest Relevant to Cost Analysis** |
| --- | --- |

The authors declare that they have no competing interests. The lead author (MIK) approached 3D Organon to request use of its software for research purposes. 3D Organon provided one Professional licence and technical support to facilitate use of the system. No financial payment was provided. The investigators had no financial relationship with 3D Organon or any competing anatomy software provider. 3D Organon had no role in the study design, data collection, data analysis, data interpretation, or manuscript writing. For the cost–consequence analysis, the licence was costed at the standard annual institutional rate (USD 299.50) to reflect real-world implementation costs rather than the research-specific procurement arrangement. The choice to use 3D Organon reflected its availability and existing use in medical education research, not a commercial arrangement. The deliberate selection of the most conservative (lowest) cadaver cost apportionment was intended to guard against any appearance of bias in favour of the digital modality.
